# High-Fidelity Long-term Whole-embryo Lineage and Fate Reconstruction by Iterative Tracking with Error Correction

**DOI:** 10.64898/2026.03.12.711203

**Published:** 2026-03-16

**Authors:** Mengfan Wang, Qinghua Zhang, Congchao Wang, Yunfeng Chi, Wei Zheng, Zeyu Mu, Xiangyu Cao, Weizhan Zhang, Boao Yang, Alexander F. Schier, Joaquin Navajas Acedo, Yinan Wan, Guoqiang Yu

**Affiliations:** 1 Department of Automation, Tsinghua University; Beijing, 100084, China.; 2 Bradley Department of Electrical and Computer Engineering, Virginia Polytechnic Institute and State University; Arlington, VA 22203, USA.; 3 IDG/McGovern Institute for Brain Research, Tsinghua University; Beijing, 100084, China.; 4 Beijing National Research Center for Information Science and Technology; Beijing 100084, China.; 5 Biozentrum, University of Basel; Basel, 4056, Switzerland.; 6 Allen Discovery Center for Cell Lineage Tracing, Seattle; Washington, 98195, United States.; 7 Lead contact

**Keywords:** lineage reconstruction, fate mapping, cell tracking, embryonic development analysis

## Abstract

Reconstructing the complete cell lineages and fate maps of a living embryo has remained a central challenge in developmental biology. Here we introduce ITEC (Iterative Tracking with Error Correction), a fully unsupervised method that automatically reconstructs the lineage of every cell in the embryo with high fidelity. ITEC was validated with manually annotated lineages on four cross-species datasets including zebrafish, mouse, and *Drosophila*. We reconstructed the developmental lineages of a zebrafish embryo from terabyte-scale data of total 18.5 million cells with an estimated accuracy of over 99.7%. With ITEC, we revealed spatiotemporal dynamics of key morphogenetic processes such as somite boundary formation, demonstrated the various patterns of spatial sorting among adjacent organs or anatomical regions, and identified associations between cellular movement and spatial transcriptomics. ITEC provides a powerful platform for retrospective fate mapping and systematic exploration of developmental dynamics at the cellular scale and single-embryo level.

## Introduction

Reconstructing the complete developmental lineage for every cell as a living embryo develops has long been considered a “holy grail” of developmental biology (1–4). Achieving this goal is critical for understanding how cells interact to form complex tissues and organs, how spatial patterning emerges during development, and how cellular diversity arises from initially homogeneous populations (5–9). With the advancement of microscopic imaging technology (3, 10–17), long-term and large-scale multidimensional datasets continue to emerge to record the dynamics of the whole embryo at the single cell level (18), establishing the foundation to achieve this goal. However, manual analysis of the whole embryo is infeasible for such terabyte-scale datasets with tens of millions of cells. Conversely, a selective manual annotation of a few cells is prone to bias and loses the systematic view of the whole embryo. Therefore, solving the bottle-neck of automatic and comprehensive lineage reconstruction would be a major advance in developmental biology.

Since the emergence of whole-embryo dynamic imaging data, there have been several efforts to computationally reconstruct the lineages and map cell fates, through either adopting generic cell tracking techniques (19–37) or designing new approaches for embryonic data (1, 38–41). Among these are three state-of-the-art methods, TGMM (1, 41), Linajea (41, 42), and Ultrack (38, 43). However, major challenges remain mainly due to two requirements: First, an extremely high accuracy is required for both detecting cells at each time point and linking cells at two adjacent time points, because any error in a reconstructed lineage along hundreds of time points will result in an erroneous, useless or misleading reconstruction. Second, the embryonic data is heterogeneous with low signal-to-noise ratio, which is a consequence of the variations in the transmissive properties of embryos, and the experimental necessity of biologically relevant conditions for embryo development.

**Fig. 1:**
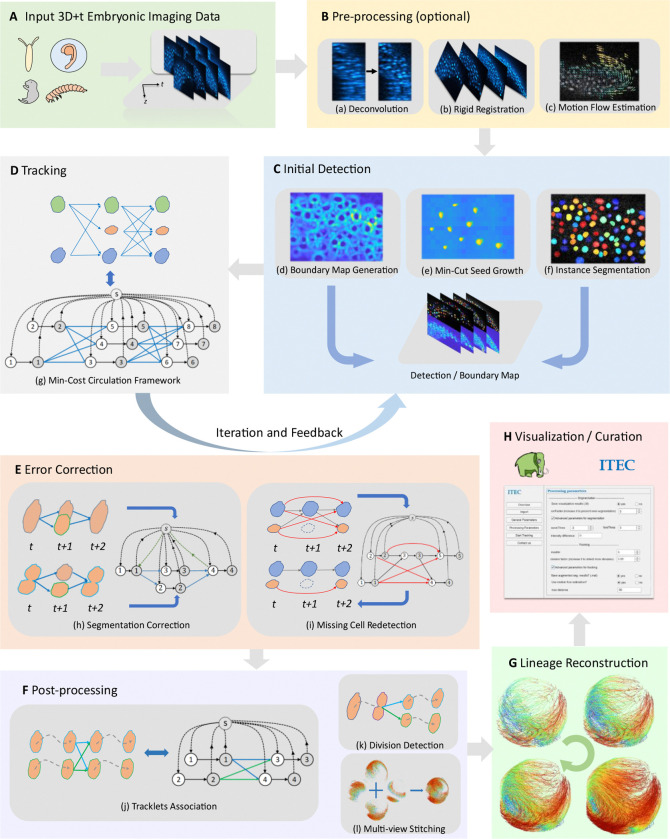
Flowchart and pipeline of ITEC. (A) A typical 3D+t input data, where each time frame consists of 3D image stacks acquired from light microscopy. ITEC supports embryonic development data of multiple species. (B) Pre-processing, which includes optional deconvolution, rigid registration, and motion flow estimation. Users can also perform individual processing steps tailored to their specific data. (C) Initial detection, utilizing high-order image information (such as in (d)) and the min-cut algorithm (such as in (e)) to achieve pixel-level instance segmentation (such as in (f)) of cells. (D) Tracking, where the problem of cell linkage across frames is modeled as a maximum a posteriori estimation problem and solved using a minimum-cost circulation framework. (E) Error correction, which includes segmentation correction module and missing cell redetection module, used to correct errors through feedback based on the last round of tracking results. (F) Post-processing, which includes tracklets association, division detection, and multi-view stitching, further enhancing the accuracy and completeness of cell linkage. (G) Lineage reconstruction. Lineages are extracted from tracking results and reformatted for the downstream analysis. (H) Visualization and curation. ITEC supports parameter tuning and execution through visualization tools, and allows visualization and curation of tracking results using Mastodon (47).

**Fig. 2:**
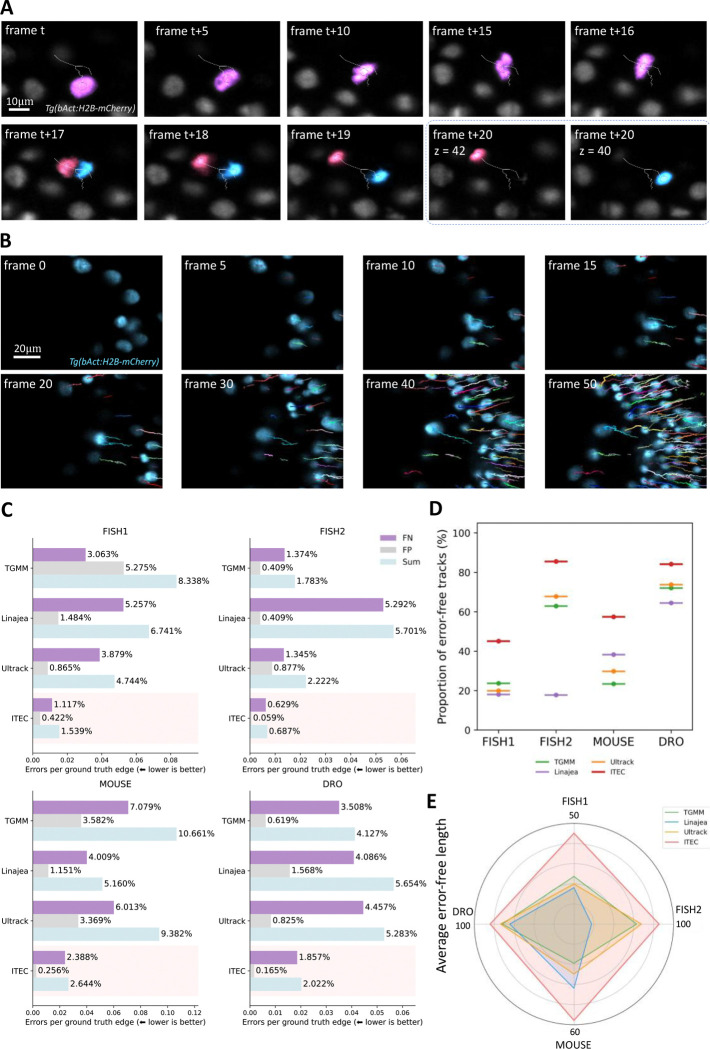
Tracking and lineage reconstruction performance of ITEC on four cross-species datasets. (A) Illustration of a typical cell track from ITEC. It consists of 20 frames. The pink cell divided in frame 17. (B) Tracks of cells from ITEC under crowded conditions. Tracks of different cells are represented in different colors. (C) Errors per ground truth edge of ITEC and peer methods. ITEC achieves the best performance with a large margin across all four datasets. (D) Proportion of error-free tracks of ITEC and peer methods. This metric represents the proportion of completely correct lineages to the total annotated lineages. (E) Average error-free length of ITEC and peer methods. This metric is the number of consecutive time points over which there are no tracking errors.

**Fig. 3:**
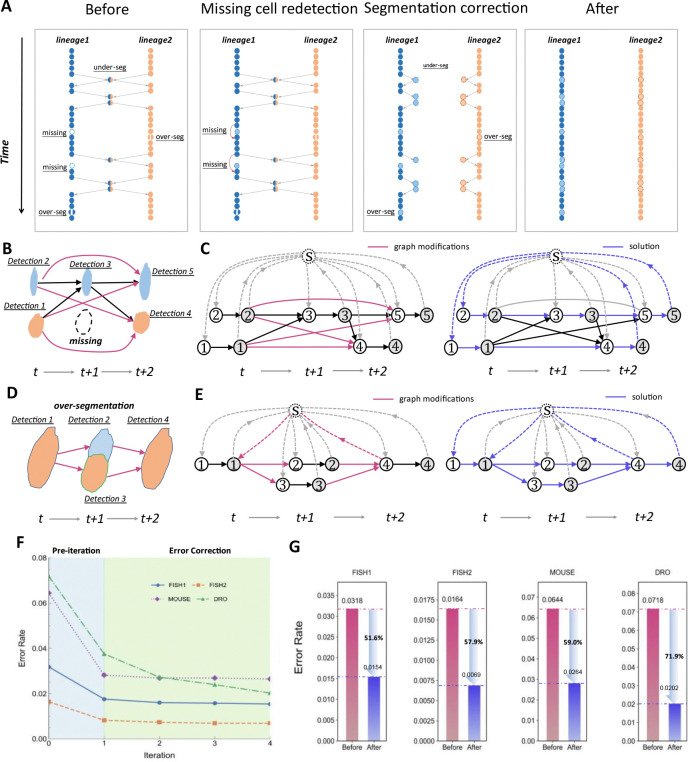
ITEC fully utilizes spatiotemporal information to iteratively correct errors. (A) Illustration of segmentation improvement through error correction module. Initially, there are 10 errors in these two lineages, including 2 missing, 2 over-segmentation, and 6 under-segmentation errors. After missing cell redetection, 2 errors are fixed, and the remaining 8 errors are resolved through segmentation correction, resulting in continuous and correct tracks. (B) Illustration of missing cell error. There are two tracks in (B). The first one is composed of detection 2, 3 and 5 and the second one consists of detection 1 and 4. A detection is missed in the second track at frame t + 1. (C) The network that allows cells to jump over some frames to be compatible with missing cells due to errors cell detection. This network design legitimates the tracking to continue from frame t to t + 2 by allowing linkages between detections beyond adjacent frames. (D) Illustration of over-segmentation error. In frame t + 1, a cell is wrongly split into two detections, while the segmentation is correct in frame t and t + 2. (E) The new design of the network allows detections to split and merge. For each detection in the (D), it is represented by two nodes in the network shown in (E). Both detection 2 and 3 can link to detection 1 or 4. With such design, the over-segmentation cells can be processed. (F) Curve of error rate vs. number of iterations. Before iteration, the error rates of FISH1, FISH2, MOUSE and DRO datasets are 0.0318, 0.0164, 0.0644, and 0.0718, respectively. After the first round of iteration, the error rates become 0.0175, 0.0082, 0.0281, and 0.0376, respectively. (G) The error rate decreased after completion of iteration. In the FISH1, FISH2, MOUSE and DRO datasets, the iterative strategy reduced the error rates by 51.6%, 57.9%, 59.0% and 71.9%, respectively.

Here, we present a full-process embryonic tracking pipeline, ITEC (Iterative Tracking with Error Correction), which achieves lineage reconstruction of whole embryos with an unprecedented accuracy (Fig. 1 and Fig. 2). ITEC exploits the special properties of the embryonic imaging data and builds upon our recent development of a new machine learning technique for data association (44). Though the heterogeneity and low signal-to-noise ratio of embryonic imaging data inherently lead to imperfections in initial cell detection and linkage, long-term spatiotemporal information precisely provides strong support to correct errors and pursue ultra-high accuracy. We developed a strategy of iterative error correction, which uses the tracking results as feedback to correct cell detection errors, and updates the tracking results based on the corrected cell detection, ultimately forming a stable closed-loop system (Fig. 3). Errors decrease continuously with iterations, which significantly improves the accuracy and robustness of tracking, particularly in scenarios with high imaging heterogeneity and low image clarity. The concept of iteratively correcting errors seems intuitive, but it was not clear how to identify and correct errors. It is our newly invented and record-setting minimum-cost circulation model (44), that enables the implementation of error correction. ITEC is user-friendly as well, as it adopts fully unsupervised techniques and requires no data labelling or human annotations. High-precision cell detection and tracking can be achieved by simply adjusting a small number of biologically meaningful parameters.

**Fig. 4:**
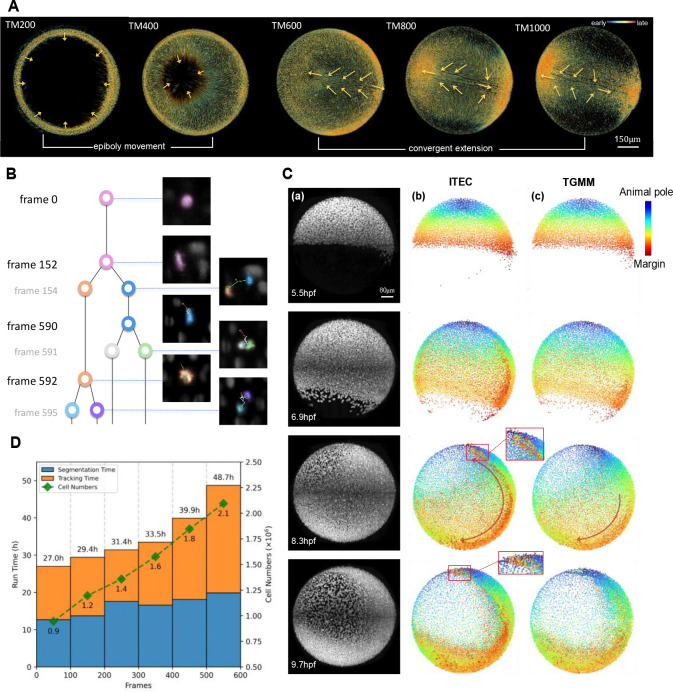
ITEC enables long-term lineage reconstruction at terabyte level. (A) Screenshots of long-term zebrafish lineage reconstruction from the Mastodon visualization platform. In the first half, cells primarily undergo epiboly movement, while in the latter half, cells undergo convergent extension. The arrows indicate the cell motion directions. (B) A typical lineage tree from ITEC. The ancestor first splits once at frame 152, and the two offspring cells split twice at frames 590 and 592, respectively. Our method can capture three divisions and accurately track the migration of all children. (C) Snapshots and the reconstruction comparison of the dataset FISH2. (a) The max projection of datasets. The rows from the first to the last show the progress of the zebrafish embryo at the time point 0, 240, 480, and 720, corresponding to 5.5, 6.9, 8.3, and 9.7 hpf, respectively. (b) The reconstruction results of ITEC. Every dot represents one detected cell in the corresponding time point, and the dot color represents the initial cell position at the time point 0. A blue dot indicates a cell originates near the animal pole, while a red dot tells the cell originates at the near the marginal zone. The purple arrows at 8.3 hpf indicates the hypoblast and the red box at 9.7 hpf indicates the hatching gland. (c) The reconstruction results of TGMM according to the same arrangement as (b). (D) Runtime variation in relation to the count of cells and frames. ITEC has an option of parallel computing to enable the distributed processing of TB-level data.

With the aid of ITEC, we automatically reconstructed lineages of over 18.5 million cells cumulatively across the entire zebrafish embryo at terabyte scale (Fig. 4). With an evaluation of a randomly selected subset of lineages spanning up to 600 time points and including over 30,000 cells, ITEC achieved an unprecedented high accuracy of over 99.7% per cell linkage and improved the lineage accuracy to 48.5% from 20% of the best peer methods. On four benchmark datasets, including two zebrafish, one mouse, and one *Drosophila* embryo development time-lapse datasets, ITEC outperformed all the three state-of-the-art methods mentioned above by a large margin across all metrics (Fig. 2). Particularly, ITEC reduced the errors per ground-truth cell linkage by at least 51.6% compared to the second-ranked method on any benchmark dataset.

**Fig. 5:**
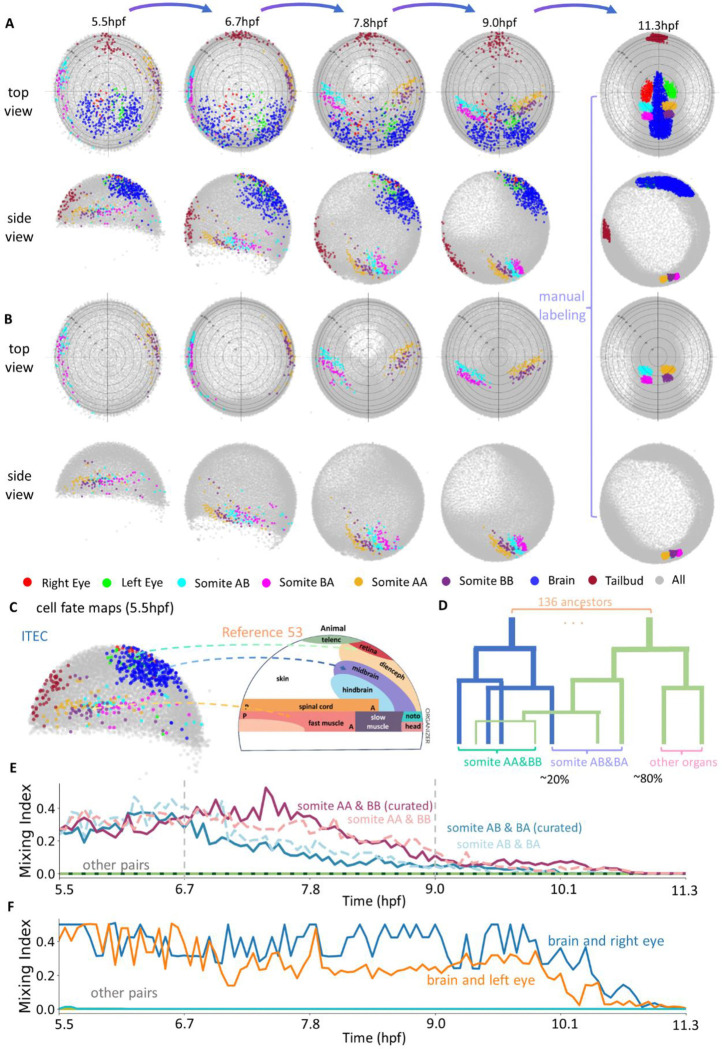
Fate mapping and mixing index analysis of zebrafish embryo development dataset based on ITEC. We manually segmented four classes of zebrafish organs: eyes, brain, somites, and tail bud, including the left and right eyes and four somites AA, AB, BA and BB, at the early organogenesis stage (11.3 hpf, the last time point of the data). (A) The top view and side view of the fate mapping results except for the last column, which visualizes the manual segmentations. The first column to the last column shows the fates of the organs at the time point 0, 200, 400, 600, and 1000, corresponding to 5.5, 6.7, 7.8, 9.0, and 11.3 hpf, respectively. (B) The top view and side view of the somite fate map according to the same arrangement as (A). (C) Comparison of a fate map of the zebrafish embryo at the start of gastrulation from (53) with the fate map generated by ITEC. (D) Schematic of ancestral and descendant lineage tree for somites AA, AB, BA and BB. (E) Mixing index for the four somites with and without curation. The ancestors of the somite AA&BB and somite AB&BA pairs gradually separate over time, whereas all other pairs such as AA vs AB, and BB vs BA remain completely unmixed. (F) Mixing index for various organ pairs in the zebrafish embryo. The ancestors of the brain/left eye and brain/right eye pairs gradually separate over time, while other pairs show almost no mixing throughout development.

**Fig. 6:**
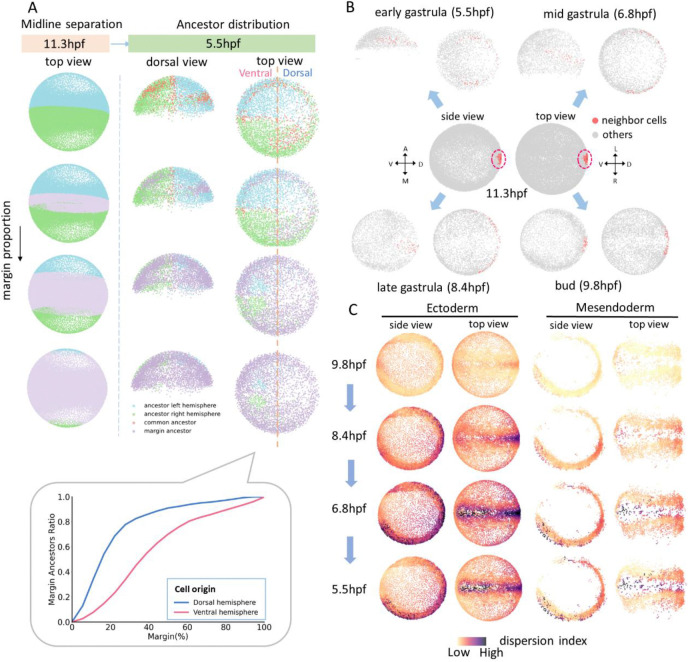
Analysis of midline formation and cell dispersion during zebrafish embryonic development. (A) Cell lineage tracing of cells bilateral to the midline in zebrafish. It shows the origin of cells bilateral to the midline and in the margin as the margin proportion increases. The bottom subfigure shows the proportion of ancestors in the margin region relative to all ancestors on the dorsal and ventral sides, respectively. (B) Ancestral distribution of a cell population at 11.3 hpf traced back to 5.5, 6.8, 8.4, and 9.8 hpf, respectively. Ancestors are represented by red scatter points. (C) Mean distance dispersion index of cells at 11.3 hpf traced back to their ancestors at 5.5, 6.8, 8.4, and 9.8 hpf, respectively. A darker color in the plot indicates a more discrete distribution of the ancestral origin of cells in that area.

**Fig. 7:**
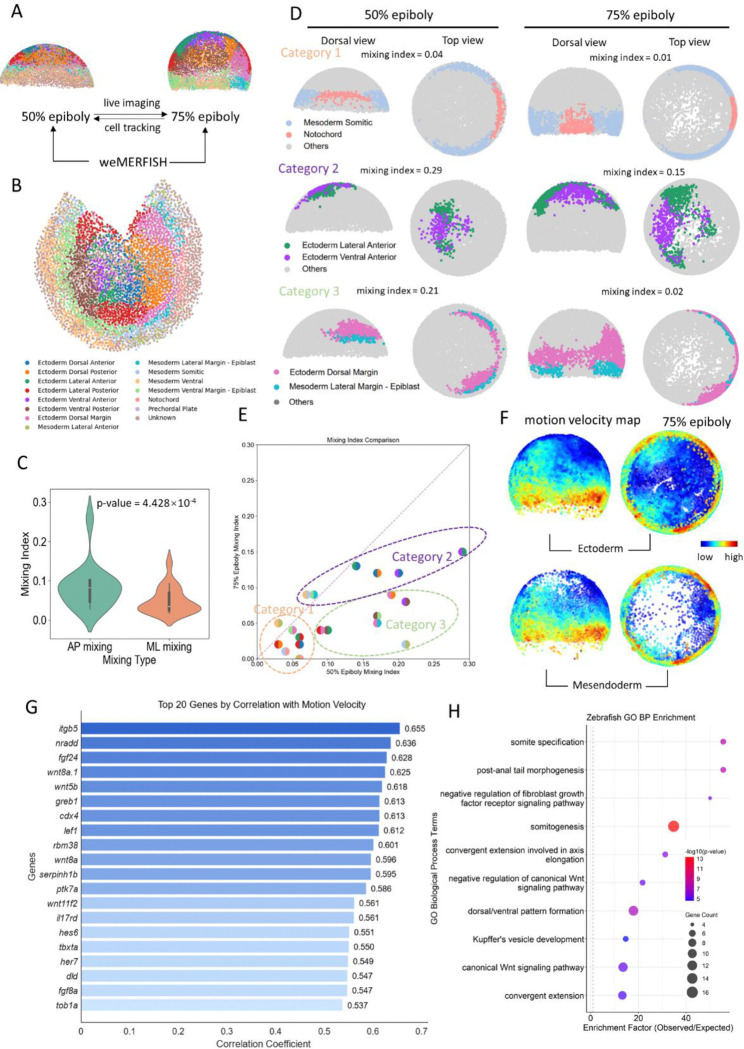
Integration of cell tracking with spatial transcriptomics weMERFISH. (A) Schematic diagram of the integration of live imaging and spatial transcriptomics. Cell association from 50% epiboly to 75% epiboly is achieved through cell tracking via live imaging, while the matching between live imaging and spatial transcriptomics is accomplished using the global optimal mapping method. (B) Distribution of the 14 regions defined by gene expression profile at 75% epiboly. (C) Wilcoxon test for cell mixing index along the anterior-posterior (AP) and medial-lateral (ML) axes. (D) Comparison of the mixing index of different mixing patterns between 50% epiboly and 75% epiboly. (E) Distribution of the mixing index at 50% epiboly and 75% epiboly. Based on the scatter of the points, we clustered them into three categories. (F) Cell migration velocity of ectoderm and mesoderm at 75% epiboly. (G) Top 20 genes most strongly and positively correlated with migration speed and their correlation coefficients. (H) GO term enrichment analysis of top 90 genes positively correlated with migration speed.

We applied ITEC to analyze the dynamic features of embryogenesis. During somite formation in zebrafish, we found that sharp somite boundaries gradually and irreversibly emerge from an initially intermixed population of progenitor cells (Fig. 5). During gastrulation, we characterized the diversity of cell mixing and rearrangements during processes such as convergence and midline formation (Fig. 6). To decipher the molecular mechanisms underlying cell behaviors, we combined embryonic lineage reconstruction with our recently developed whole-mount spatial transcriptomics (9) (Fig. 7). In summary, ITEC transforms cell tracking from an error-prone, fragmented process into a robust end-to-end solution, thereby turning large-scale and long-term embryo lineage reconstruction from a burdensome task into a practical and routinely feasible approach for biological research.

## Results

### Unsupervised full-process pipeline of Iterative Tracking with Error Correction (ITEC)

Long-term tracking of embryonic cells demands extreme precision, imposing high requirements on the collaboration between processes such as cell segmentation and cell linkage. Iterative Tracking with Error Correction (ITEC) is a high-fidelity and full-process cell tracking pipeline designed for embryonic lineage reconstruction and fate mapping, which is potentially applicable for the generic cell tracking tasks but not comprehensively tested yet. It is an end-to-end process, covering the entire procedure from raw data pre-processing to the final reconstruction of lineages, with no user intervention required in between. In addition, it is entirely based on unsupervised learning and does not require any labeling. For more technical details, please refer to Online methods.

The flowchart of ITEC is shown in Fig. 1. First, we provide users with optional pre-processing operations to improve image quality and correct imaging artifacts, including deconvolution (45), rigid registration and motion flow estimation for non-rigid deformation (27) (Fig. 1B). These steps allow meaningful biological signals to be enhanced from imaging data with heterogeneous cell activity limited by noise and resolution. Then we perform initial cell detection by applying principal curvatures to mimic human perception (Fig. 1C, Fig. S2 and Fig. S4) (46). We combine curvature maps under different scale filtering and utilize robust statistical tests to effectively exclude background regions and counteract data heterogeneity. The boundary refinement problem is modeled as the min-cut graph problem to ensure pixel-level instance segmentation. After the initial detection, we transform the cell linkage problem into a global discrete optimization problem and build the minimum-cost circulation framework that can efficiently handle millions of cells (Fig. 1D).

Subsequently, we use an iterative error correction strategy to further refine the segmentation and cell linkage results based on the existing tracking results, making full use of the rich spatiotemporal information in long-term lineage reconstruction (Fig. 1E). This strategy involves multiple iterations, and results gradually become stable in the process of segmentation correction and recovering the missing cells. To ensure the continuity of the tracks and consistency with biological hypotheses, we perform tracklets association, division detection, and multi-view stitching (Fig. 1F). For user convenience, we also design an interactive interface and integrate our tracking results with Mastodon (47), a Fiji (48) plugin for cell tracking analysis (Fig. 1H). Among all the technical innovations in developing the ITEC pipeline, the iterative error correction strategy and the minimum-cost circulation modeling of data association were the two most effective, which are further discussed and evaluated with ablation experiments in the next sections.

### ITEC outperformed peer methods on four cross-species datasets

To comprehensively compare our method with peer methods, we conducted experiments against ITEC and three state-of-the-art peer methods TGMM (1, 39), Linajea (41, 42), and Ultrack (38, 43). These methods respectively adopt statistical approaches, utilize deep learning techniques, and integrate segmentation and linking, reflecting the latest advancements in the field of cell tracking. More details about the peer methods can be found in Table S1. We then selected four cross-species datasets, including zebrafish, mouse and *Drosophila* embryo (FISH1, FISH2, MOUSE, and DRO) (Table S2) and constructed the ground truth by combining public annotations with our manual annotations. The annotations resulted in total of 44,555 ground-truth edge linkages. Characterized by diverse signal-to-noise ratio, temporal resolutions, spatial resolutions, and cell densities, performances on these datasets shall be a good indication of the robustness and generality of these tracking methods in real applications (Fig. 2A, Fig. 2B, Fig. S2A and Fig. S2B). For the definitions of the evaluation metrics, please see Fig. S1 and Supplementary Materials.

We first evaluated the accuracy of each edge linkage between adjacent frames. ITEC achieved a lead by a large margin across all four datasets (Fig. 2C). Its sum errors per ground truth edge (error rate) on FISH1, FISH2, MOUSE, and DRO were 32.44%, 38.53%, 51.24%, and 48.99% of that of the second-ranked method, respectively. Among these, ITEC gained a more significant lead on the two zebrafish embryo datasets FISH1 and FISH2, primarily due to the higher spatial continuity and stronger inter-frame correlation information. For the mouse embryo dataset, the opacity of mouse embryos and the long imaging intervals required for long-term imaging resulted in an error rate of ITEC exceeding 2.5%. Excluding ITEC, none of the three peer methods dominated the other two. TGMM worked well on FISH2 and DRO datasets, Ultrack suited for FISH1 dataset, and Linajea worked best in MOUSE dataset. Notably, during the evaluation process, we only need to set fundamental parameters such as grayscale, size, and filtering parameters, without making any modifications to the method ITEC itself. This indicates that our method exhibits high robustness and can meet the analytical needs of various model organisms used by the community.

While some lineages are difficult to reconstruct, leading to a lot of linkage errors, others are easier to follow, with small or no errors, and result in better lineage accuracy than expected through independence assumption. In order to quantify the performance on long-term lineage reconstruction, we selected *proportion of error-free tracks* and *average error-free length* to evaluate the continuity and reliability of tracks. Error-free tracks are complete paths along which cells can be followed correctly, while error-free length indicates the maximum number of frames a method can track correctly in a single, complete path. The reason for choosing these two metrics lies in the fact that biologists are interested in the tracking of cells during long-term migration. Thus, these metrics better reflect the feasibility of cell tracking that holds biological value compared to metrics such as edge linkage accuracy. We calculated the average of error-free length across all lineages in the ground truth to assess the continuity of the reconstructed lineages. In addition, we defined the proportion of error-free tracks out of the total number of lineages in the ground truth to measure the ability for long-term cell migration tracking. Experiments demonstrated that our method also achieved the best fidelity in lineage-scale evaluation (Fig. 2D, Fig. 2E and Fig. S3). In the FISH2 dataset, 85.5% of lineages remained completely accurate over 100 frames, compared to the next best of Ultrack being 67.7%. In the FISH1 dataset with 192 frames, longer imaging intervals and faster developing speed, ITEC achieved complete accuracy in 45.1% of lineages versus 23.9% obtained by the next best approach, TGMM.

### Iterative error correction strategy improves the overall tracking accuracy

A major challenge in high-fidelity lineage reconstruction lies in the accumulation of errors. On the one hand, from the perspective of long-term cell tracking, errors accumulate over time because one error in a long track can render the entire track invalid. On the other hand, errors can propagate within a certain spatial range, leading to a chain reaction of incorrect linkages between surrounding cells. The vast majority of these errors stemmed from the instability of segmentation and seemed to be unavoidable owing to the low and heterogeneous image quality. It is hard to achieve high image quality due to factors such as uneven illumination, variations in the transmissive properties of embryos, and limitations of imaging equipment (Fig. S2A). Moreover, to minimize toxicity, the experiments are often planned to have a signal-to-noise ratio as low as possible.

Interestingly, when annotating a time-lapsed image dataset, human experts can overcome these limitations by leveraging the rich spatiotemporal information residing in the data. Therefore, we designed an iterative error correction strategy, expecting an initial lineage reconstruction can be leveraged to identify and correct errors. For example, if there are cells at the same location in both frame *t* and frame *t*+2, there is a high probability of a cell being missed in frame *t*+1 (Fig. 3B). If there is only one cell in consecutive frames at a certain position, and in one frame it is detected as two cells, then that frame is likely to have undergone over-segmentation (Fig. 3D). We can leverage the cell segmentation and linkage results within a time window, combined with prior information such as cell size and spatial relationships, to infer the most probable scenarios, thereby compensating for missing cells or resolving segmentation errors. ITEC is capable of multiple iterations, in which the missing cell recovery and segmentation correction are performed sequentially in each iteration, gradually reducing errors during the iteration (Fig. 3A).

To validate the effectiveness of the iterative error correction strategy, we conducted ablation experiments: with only one round of error correction, the error rates of the FISH1, FISH2, MOUSE and DRO datasets were reduced by 45.0%, 50.0%, 56.4%, and 47.7% of the original error rates, respectively (Fig. 3F). After 3 iterations, the error rates tended to stabilize, and the final error rates on the four datasets were 1.54%, 0.69%, 2.64%, and 2.02%, respectively, which were 51.6%, 57.9%, 59.0%, and 71.9% lower than the original error rates, respectively. Among these, the DRO dataset has the greatest effect with errors dropped 3.5-fold. We found that the first iteration of error correction is usually the most effective. Although there was almost no effect after 3 iterations, we set 5 iterations as the default parameter value in our program to safeguard more difficult scenarios. Through an iterative error correction strategy guided by spatiotemporal information, ITEC improves greatly the accuracy of tracking and lineage reconstruction.

### Minimum-cost circulation model facilitates the error correction and helps maintain the integrity of tracks

While iterative error correction is conceptually straightforward, it is technically difficult to implement, due to the challenge in identifying errors or the concern of increased computational costs. We addressed both issues at the same time by adopting our recently developed computationally efficient and flexible model, the minimum-cost circulation model (44) (Fig. S5). Firstly, the model set a new record of computational complexity, which was further supported by the empirical evidence that it achieved an average increase in speed of 53 to 1192 times over three peer algorithms across multiple datasets (44). This improvement in efficiency relieved us from concerns about computation cost. Secondly, we introduced further modifications to the original minimum-cost circulation model to identify the localizations of the erroneous candidates for the error correction module. (1) To address the error of missing detections, frame-skipping connections were added (Fig. 3B and Fig. 3C). The model can tell whether a frame-skipping connection is turned on to fit the observed data. When the connection is turned on, it suggests there is a missing cell between the two connected cells. Then a module of detecting missing cells is called on to redetect them and splice originally broken tracks into continuous ones. (2) To tackle over-segmentation and under-segmentation errors, we add two arcs to the model and permit one-to-two and two-to-one matchings at specific time points (Fig. 3D and Fig. 3E). If the cost of splitting or merging cells is lower than that of the original segmentation result, and other biological or probabilistic constraints are satisfied, a decision is made to correct the segmentation result.

To enhance track integrity, the minimum-cost circulation model was further modified to allow for cell motion with large displacement. Due to individual cell differences and biological activities, some cells or cell populations have large motion within specific time periods (Fig. S8A). Therefore, we initially make a conservative estimate and only use high-confidence linkages to determine error corrections. Here, the tracking results obtained with the stringent linking standard are named tracklets. Eventually, large motion associations become increasingly reliable and more relaxed linkages can be tolerated. Finishing all the iterations of error correction, we abstract the existing tracklets into individual nodes (Fig. S8B) and apply the minimum-cost circulation model again to allow for a wider range of motions. This trick enables the implementation of error correction strategy and the merging of numerous broken tracks while ensuring high accuracy.

### ITEC enables long-term lineage reconstruction at terabyte level

Time-lapse 3D imaging datasets of embryonic development are often at terabyte-scale (18). In order to maintain accurate tracking during long periods of cell migration and division. Due to the accumulation of tracking errors, only high-fidelity tracking can lead to conclusions with practical biological significance. To our knowledge, current tracking methods often lack the capability to handle large-scale data, fail to output results in complex scenarios, have excessively long runtimes, or exhibit a high error rate during long-term tracking.

To test the capability of ITEC in long-term lineage reconstruction, we used a dataset consisting of 1001 time points (39). This dataset captures 16.7 hours of whole-embryo development in zebrafish, with a total data size of 1.5 TB (1001×253×1792×1818 voxels, T×Z×Y×X). We aligned the time window with the absolute developmental time, mapping it to the 5.5–11.3 hpf period (49). We ran our pipeline on this dataset in a distributed manner, dividing the 1001 frames into several batches with a certain overlap between adjacent batches. Subsequently, we associated the results from each batch using the nearest neighbor principle. To test the accuracy of these lineages, we randomly selected 31 cells at the first time point and then manually curated the corresponding lineages for the first 600 frames, which contained a total of 9.0 million cell instances with about 8,000 cells at the first time point and 21,000 cells at the end. Among over 30,000 linkage edges and a similar number of cell instances, a total of 91 errors were identified, which means the linkage accuracy of our method reached 99.7%. More notably, 15 lineages, that is, 48.4% of lineages, were error-free in the analysis spanning 600 frames (each lineage typically contains 1 to 3 divisions). Fig. 4A and Fig. 4B show the results obtained by ITEC.

To the best of our knowledge, it is the first time that the lineages of the whole-embryo containing up to 21 thousand cells at one time point and spanning 600 time frames can be fully reconstructed at the level of around 50% error-free and complete lineages. In contrast, the best peer methods are estimated to achieve only a 20% error-free rate. It is worth pointing out that as 10 seconds are usually needed to annotate one cell instance such an embryo with total 9 million cell instances would take 25,000 manual hours (more than 1,000 days) to be fully annotated. In contrast, ITEC can complete the same analysis in 8.7 days in a fully serial manner with a single processor and achieve results comparable to human labeling (Fig. 4D). If distributed operation is adopted, the analysis time will be shortened to 2–3 days.

### Capturing complex movements during embryonic development

Based on the long-term, high-fidelity tracking performance, we expected ITEC to be effective in capturing morphogenetic events. Analysis of the tracks during zebrafish embryogenesis (Fig. 4A) reveal epiboly movement, the movement of cells towards the vegetal pole, and convergent extension, the intercalary movement of cells towards the dorsal side (50). To further investigate whether ITEC is capable of capturing developmental dynamics at key stages, we analyzed internalization movements. Internalization is the defining cell motion observed during gastrulation. It is one of the most difficult motions for tracking, because cells ingress, undergo large-scale movements and rearrangements, and become less accessible for imaging (50). Starting from 50% epiboly at 5.5 hpf, the zebrafish blastoderm margin moves and folds inwards, generating an outer and inner layer, the epiblast and hypoblast, respectively (51). Cells in epiblast and hypoblast have opposite motion directions, move rapidly, and change neighbors, making cell tracking very challenging. Since involution takes place during early stages of embryogenesis, any tracking mistake at this stage carries forward to later stages, resulting in erroneous reconstructions.

Even with such a challenging dataset, ITEC captured internalization (Fig. 4C and Movie S3). We compared our approach with the classic algorithm TGMM (1, 39), which was especially designed for embryonic cell tracking. The most significant difference can be observed in the box in Fig. 4C at 8.3 hpf. Red cells represent involuted cells in the hypoblast, whose color indicates that their origins were from the blastoderm margin as tracked by ITEC. In contrast, TGMM cannot track these margin cells correctly and the internalization process was clearly missed (52). These results show that ITEC can reconstruct cell lineages over long distances and through rapid and complex cell movements.

### ITEC enables accurate fate mapping of multiple tissues at the single-embryo level

High-accuracy, long-term lineage reconstruction based on *in vivo* imaging provides a powerful approach for revealing complete fate maps, in which the earlier location of all the cells in the same embryo can be linked to its later position and fate. To test the potential of ITEC in fate mapping, we manually annotated the positions of several organs during zebrafish embryonic development at 11.3 hpf and then applied ITEC to trace back to their ancestors retrospectively to 5.5 hpf (Fig. 5A). The result demonstrated a high degree of consistency with existing fate maps reconstructed from *in vivo* clonal tracing methods (53, 54) (Fig. 5C). The positions of progenitors of the brain, eyes, and somites (future muscles) align well with the reported progenitor location in existing maps (51, 54–57). Another evidence that validates the correctness of ITEC is the accurate backtracking of somites. The four tracked somites (namely AA, AB, BA, and BB) are located in close proximity at 11.3 hpf. When mapped to 5.5 hpf, AA/AB and BA/BB are clearly assigned to the left and right hemispheres of the embryo without any mistake (Fig. 5B).

To further validate the capability of ITEC in fate mapping, we manually curated the lineages of 293 labeled descendants in somites at 11.3 hpf (Fig. 5D), where over 192 thousand cells were involved in total and 17 descendants at 11.3hpf were excluded due to poor imaging quality. For our curation process, we worked backwards from the end timepoint of the dataset, paying close attention to how critical events like cell division and large displacements were handled. On average, it took about 0.8 hours to curate a single cell’s entire 1001-time-point lineage, amounting to a total of roughly 220 hours for the entire curation. As a comparison, manually mapping all of the 276 descendants would require about 800 hours. After curation, we found that these somites originated from a total of 136 ancestors (Fig. 5E) at 5.5 hpf. Without curation, the automatic fate mapping of the 276 descendants identified 94 ancestors, of which 65% are the correct ancestors. Such a good fidelity enabled drawing biologically meaningful conclusion based on automatic fate mapping, which was confirmed in the next section.

Compared to the conventional clonal analysis, ITEC enables the fate mapping of multiple tissues or organs on the same embryo, which empowers further analysis of the boundary forming of adjacent tissues and the relative developing time among different organs, as demonstrated in the next sections.

### ITEC reveals the dynamic formation process of boundaries from a mixed population of cells

Time-lapse imaging data, combined with an efficient tracking method, can reveal how intermingled cells segregate into defined structures and territories. Somites are the paired blocks of paraxial mesoderm that will become the adult muscles. During somitogenesis, the unsegmented paraxial mesoderm progressively segments into bilaterally symmetrical somites with defined boundaries (58, 59). To test the capability of ITEC in exploring cell intermingling and segregation, we analyzed somite formation. ITEC revealed the entire dynamic process of the formation of somites in the anterior part of the animal (Fig. 5B and Movie S5). The paraxial mesoderm of zebrafish derives from two symmetrical territories close to the margin that converge toward the margin, internalize and migrate along the anterior-posterior axis. Interestingly, ITEC allowed us to determine that at 5.5 hpf, progenitor cells that will give rise to separate somites exist in an intermixed state without discernible boundaries. In order to accurately quantify the formation process of the somite boundary, we fit the classification hyperplane to the somite pairs at each timepoint and defined the average misclassification ratio of the two classes as the mixing index (Fig. 5E). Starting from 6.7 hpf, the mixing index gradually decreases, indicating that in ipsilateral somites AB and BA, as well as AA and BB, cell populations become gradually separated, marking the initial formation of somite boundaries. We notice that although somites are bilaterial they are not completely symmetric. Somites AB and BA segregated earlier and faster than somites AA and BB. Until 9.0 hpf, the mixing index decreases to a very low level and fluctuates slightly, indicating that boundary structures are established as interfacial gaps emerge at their contact zones. Overall, starting at 6.7 hpf, the mixing index shows a monotonic downward trend, indicating the transition of somite progenitors from a mixed to a segregated state. Notably, measuring the mixing index without human curation led to the same conclusion.

We extended the analysis of the mixing index to brain and eyes (Fig. 5F). The ancestors of the brain and eyes were heavily intermingled at 5.5 hpf until having a more distinct boundary at 10.1 hpf, and significantly separated at 11.3 hpf, agreeing with previous reports (54). In addition, the left and right eyes exhibited a similar ipsilateral mixing pattern as the bilateral somites, with no contralateral intermixing from beginning to end. Given that the distance between the left and right eye progenitors was very close at 5.5 hpf, these results highlight the accuracy of ITEC in revealing the continuous spatiotemporal features of organ development and the gradual transition of cells from a mixed to a segregated state.

### Revealing the heterogeneity and distribution pattern of multiple regional origins

The developmental origin of midline structures – notochord, floor plate, spinal cord and more generally the body axis – and the diversity of cell behaviors that give rise to the embryonic midline have long been under debate (60, 61). Leveraging whole-embryo lineage tracing with ITEC, we revisited this classical problem by directly linking early progenitor positions to their later midline contributions.

We identified the midline of zebrafish embryo at 11.3 hpf and traced back the progenitor cells at 50% epiboly that contribute to the left and right hemispheres as well as to common bilateral progenitors. We found that these bilateral progenitor cells were symmetrically distributed along the midline formed by the shield, and originated from regions further away from the midline at the neural plate border (Fig. 6A). To analyze spatial asymmetries, we expanded the midline domain at 11.3 hpf outward to define three regions – the midline, left and right territories – and examined the distribution of their ancestral cells across the dorsal and ventral hemispheres. Cells originating from the dorsal hemisphere contributed proportionally more to the midline than those from the ventral hemisphere (Fig. 6A), and this asymmetry persisted as the midline domain expanded. This bias likely reflects the fact that dorsal cells approach the midline more directly through convergence movements during gastrulation.

Subsequently, we examined whether tissues and organs arise through the coherent migration of cell populations or through the reorganization of cells originating from distinct locations. To address this question, we selected cells and their neighboring populations at 11.3 hpf and traced their ancestors back to the 5.5 (early gastrula), 6.8 (mid gastrula), 8.4 (late gastrula), and 9.8 (bud) hpf stages, respectively. As shown in Fig. 6B, when a group of tightly arranged cells near the midline was selected, their ancestors were found to originate almost equally from the left and right hemispheres, supporting the hypothesis of cell reorganization. Between 5.5 hpf to 6.8 hpf, these ancestral cells underwent vegetal movement during epiboly and became increasingly dispersed on both sides of the embryo. Immediately afterward, during convergence, cells from both hemispheres aggregated toward the dorsal midline, ultimately forming the compact cell cluster observed at 11.3 hpf.

At 50% epiboly, the blueprint for the different germ layers of a zebrafish embryo is established (50), and secondary movements begin. We wondered if the different germ layers would show different speeds or cell dispersion capacities from the midline across gastrulation. We quantified these cell movement events using a mean distance dispersion index, which measures the degree of ancestral dispersion based on the average value of pairwise distances among the traced cells (Fig. 6C). The dispersion index of cells near the ectodermal midline was significantly higher than that of cells in other regions, indicating a broad and heterogeneous origin of cells near the midline. Notably, dispersion peaked near the end of epiboly, when cells were maximally distributed across the spherical embryo surface. Together, these analyses provide a novel perspective for verifying and quantitatively analyzing challenging embryonic development processes, including cell mixing and rearrangement, and enable the systematic revelation of the origin and overall behavior of cell populations across germ layers.

### Integration of ITEC with spatial transcriptomics identified correlation between cell dynamics and molecular programs

During embryonic development, the spatiotemporal expression of genes provides crucial insights into cell behaviors and fate decisions, but static measurements capture only snapshots at specific time points. In contrast, *in vivo* imaging reveals dynamic processes such as cell migration and differentiation, yet lacks comprehensive molecular information about the underlying gene expression programs. To bridge these complementary approaches, we applied our recently developed technique, weMERFISH, a whole-embryo spatial transcriptomics platform based on multiplexed error-robust fluorescent in-situ hybridization (9), which captures gene expression patterns across the entire embryo. In our previous work, we developed MERFISH-FATE, which integrated whole-embryo spatial transcriptomics with *in vivo* cell tracking to reveal how gene expression patterns evolve through morphogenetic movements during gastrulation, and showed that similar spatial patterns can arise through distinct transcriptional dynamics. Building on this framework, we characterized in more details the correlation of gene expression with multiple morphodynamic features. We integrated the spatial coordinates of weMERFISH data with ITEC by applying a globally optimal matching algorithm (Fig. 7A) to align corresponding regions between the 50% epiboly and 75% epiboly stages. Because of embryo-to-embryo variability, this alignment represents an approximate correspondence of tissue regions rather than single-cell matches, enabling the exploration of how gene expression dynamics relate to morphogenetic cell movements at the tissue and organ levels.

We used the lineage reconstruction results to calculate a cell migration velocity map at the 75% epiboly stage (Fig. 7F). Cells located at the margin exhibited the highest migration speeds, with dorsal cells showing the fastest movements consistent with convergence during gastrulation. To link cell dynamics with molecular programs, we computed the correlation coefficients between the local migration velocity and gene expression levels (Fig. S10). Fig. 7G shows the top ten most correlated genes, which include *itgb5*, *nradd*, *fgf24*, *wnt8a.1*, and *wnt5b*. We then performed Gene Ontology (GO) enrichment analysis on genes with the strongest correlations (Fig. 7H). The most significantly enriched categories included somite and tail development, as well as components of Wnt and Planar Cell Polarity (PCP) (*wnt8a, lef1, wnt5b, wnt11f2, ptk7a*) (62–64), Fgf (*fgf8a, fgf24*) (65) and Notch (*dld*) (66) signaling pathways, suggesting that these signaling cascades might be linked to the characteristic fast motion of cells during convergent extension.

The local coherence of cell velocities across different regions reflects the tissue mechanical properties such as fluidity index (67, 68). To relate these properties to gene expression, we calculated the local variance of velocity within neighborhood of 50 cells (Fig. S12) and identified genes whose spatial expression patterns resembled the variance map (Fig. S11). We found that the spatial heterogeneity in cell movement is associated with developmental programs that control mesodermal remodeling, vascular morphogenesis, and axis patterning, such as genes *hmcn2.1*, *nid2a* and *tcf7*, highlighting increased fluidity in the mesendodermal tissues (69) and the molecular basis of mechanical and behavioral diversity across the embryo.

The integration of ITEC with spatial transcriptomics enables direct connections to be drawn between gene expression patterns and cell dynamics, helping establish a solid foundation to elucidate the spatiotemporal mechanisms underlying cell behavior and providing a new perspective for studying developmental processes.

### Molecularly defined regions are asynchronously formed

Using the cell mapping relationships derived from live cell tracking, we projected the cell-type identities at the 75% epiboly back onto the 50% epiboly stage (Fig. 7B) to investigate the mechanisms underlying boundary formation between transcription-defined regions. This process is similar to fate mapping, with the key difference lying in the source of the region annotations, which were obtained through weMERFISH spatial transcriptomics (9). Among the 14 regions with class labels, we analyzed the mixing at the boundaries of 22 region pairs, excluding non-adjacent region pairs and those belonging to the different layers. We fitted a separating hyperplane for each pair of regions and defined a mixing index as the sum of the proportions of misclassified cells on both sides of the hyperplane.

Through clustering the distribution of the mixing index at the two epiboly stages, three distinct mixing patterns emerged (Fig. 7E). One example for each category was shown in Fig. 7D and more examples were detailed in the supplementary figures Fig. S13-S15. We found that 9 region pairs maintained relatively clear boundaries at both the 50% and 75% epiboly stages (Fig. S13). For example, somitic mesoderm and notochord clusters maintained clear boundaries at both stages, consistent with the conclusions we previously obtained (9) (Fig. 7D). Additionally, 7 region pairs exhibited strong mixing at 50% epiboly, but the intermixed cells segregated to molecularly and anatomically distinct clusters at 75% epiboly (Fig. S14), similar to the patterns we observed in somite segment formation at much later stage of 11.3 hpf (Fig. 5F). For example, lateral anterior mesoderm and somitic mesoderm cells were much intermingled at 50% epiboly but became completely segregated at 75% epiboly (Fig. 7D). Furthermore, 6 region pairs remained on a relatively mixed level at 50% through 75% epiboly stages (Fig. S15). Note that the non-separated boundaries for these region pairs at 75% epiboly may be partly due to the inaccuracy from the registration between individual embryos.

We were also interested in the directionality of mixing at the boundaries, so we further analyzed the directionality of cell mixing across all regions by quantifying the cell mixing indices along the anterior-posterior (AP) and medial-lateral (ML) axes, respectively (Fig. 7C). This analysis showed that the cell mixing along the AP direction was significantly greater than that along the ML axis (Wilcoxon test, p=4.428×10^−4^). All these results demonstrated that the molecularly and anatomically defined regions were asynchronized and formed at different epiboly stages, likely reflecting different underlying mechanisms.

## Discussion

Accurate lineage reconstruction in 3D time-lapse embryonic development dataset has long been a major challenge in the field of developmental biology. Due to factors such as the heterogeneity of image quality, the vast scale of data, and the extremely high requirements for accuracy, current mainstream methods have not yet truly solved this problem. To address these challenges, we propose ITEC, a cutting-edge pipeline designed for embryo tracking in live-imaging microscopy dataset, with the ultimate goal of reconstructing complete and accurate lineages. We fully exploit the spatiotemporal information contained in the multi-dimensional data, and skillfully utilize our novel error correction strategies. This enables mutual promotion between cell detection and linkage, forming a stable closed-loop feedback system, thereby significantly enhancing the accuracy and robustness.

ITEC has achieved a significant lead in tracking accuracy compared to the state-of-the-art methods. We validate our approach on embryonic development datasets of common model organisms such as zebrafish, mouse, and *Drosophila*, and ITEC consistently yields the best performance—with an average error rate reduction of 58.5% compared to the second-ranked method. Our method also achieves the highest lineage continuity and overall accuracy in the evaluation of long-term lineages. The lineage reconstruction task imposes extremely high standard on accuracy: a single error among hundreds or thousands of frames can lead to mistakes in cell lineage tracing and fate mapping, thereby rendering the results unreliable. We believe that ITEC, with an unprecedentedly low error rate, has made this once-impossible task feasible. It is expected to facilitate the emergence of a new paradigm in developmental biology research of both model organisms and *in vitro* models such as organoids and embryo-like structures.

Then, what exactly enables ITEC to achieve such high accuracy? We attribute this to the full utilization of spatiotemporal information. While other methods also leverage temporal consistency to a certain extent, our method takes the thorough exploration and refined the feedback of this information. By analyzing and examining numerous cases where the tracking errors occur, we have designed a comprehensive error correction module. This module, when integrated with a minimum-cost circulation framework, identifies potential errors from the initial tracking results, corrects them, and achieves stability through multiple iterations.

We validated the performance of our method on data spanning 1,001 frames and terabyte scale. Furthermore, we utilized these results to conduct analyses of fate mapping and the findings are consistent with existing advancements in developmental biology, verifying the accuracy of our method. Previous research used clonal analysis by single-cell transplantation or fluorescent labeling to analyze cell fate by a sum of several embryos (51, 54–57). However powerful, this method can miss fuzzy tissue boundaries or will miss the fate and lineage relationships of the unlabeled neighbors. Using ITEC, we could investigate the formation process of zebrafish somite development from a dynamic perspective and explored the origin of midline structures *in toto*, providing biologists with a novel method that combines cell-level tracking with organ-level research. Additionally, we quantified a series of important kinetic parameters during embryonic development, such as the dispersion index of cell origin, cell migration speed, and variance from a quantitative viewpoint. We also integrated *in vivo* imaging with spatial transcriptomics, and based on this, explored issues including organ boundary formation and the relationship between cell migration and gene expression. These studies strongly demonstrate that our method not only achieves excellent performance in terms of tracking accuracy but also holds great potential for advancing biological research.

To provide convenience to the broader community, ITEC strives to be user-friendly. Therefore, during development, we retained parameters with biological or image-related significance for users to adjust, and concretized complex principles into several key parameters. In addition, ITEC allows users to adjust parameters via a visual interface and integrates tracking results with Mastodon (47), a large-scale cell tracking platform, facilitating users’ downstream analysis.

Despite its advantages, ITEC still has some limitations. First, ITEC’s runtime can be further optimized: for large-scale datasets, if users do not adopt a distributed computing strategy or have no access to parallel computing solutions, the analysis time will be relatively long, which may affect the user experience. Going forward, we will improve operational efficiency and user experience by designing better parallel strategies and optimizing the underlying time-consuming modules.

Additionally, our method was originally designed for the identification and tracking of nucleus-labeled embryonic cells. It would be suboptimal when segmenting cells with diverse morphologies—such as polyhedral, columnar, or spindle-shaped cells—or cells with heavily uneven internal expression. Although certain corrections can be achieved through the error correction module, we aim to expand the versatility of the ITEC framework in future work, thereby enabling the segmentation and tracking of other cell types.

In the future, we also aim to integrate embryo tracking with other biological technologies (9, 70–72). For instance, while we currently use nuclear channels for tracking, integrating with membrane channels is expected to enable the exploration of morphological dynamics. Additionally, the deeper integration of embryo tracking and spatial transcriptomics on the same embryo is expected to reveal the correlation between developmental rhythms and gene expression, thereby advancing our understanding of embryonic development from multiple perspectives.

## Supplementary Material

Supplement 1

## Data Availability

The tracking evaluation results of ITEC and peer methods in this paper, as well as the ground truth we used are shared through Mendeley with the dataset identifier https://dx.doi.org/10.17632/tg55phtk4r.1. The FISH1 (9) and FISH2 (41) data will be shared by the lead contact upon request due to the large amount of data. The MOUSE (1) data can be downloaded from https://idr.openmicroscopy.org/webclient/?show=project-502. The weMERFISH (9) website: https://schier.merfisheyes.com/.

## Code availability:

All original code has been deposited at https://github.com/yu-lab-vt/ITEC.
