# Supplementary material for: High-Fidelity Long-term Whole-embryo Lineage and Fate Reconstruction by Iterative Tracking with Error Correction
Source: bioRxiv. 2026 Mar 16:2026.03.12.711203. Preprint. [Version 1] doi: 10.64898/2026.03.12.711203 (PMC13015363; doi:10.64898/2026.03.12.711203)
Supplement: Supplement 1 [file NIHPP2026.03.12.711203v1-supplement-1.pdf]

bioRxiv preprint doi: https://doi.org/10.64898/2026.03.12.711203; this version posted March 16, 2026. The copyright holder for this preprint (which was not certified by peer review) is the author/funder, who has granted bioRxiv a license to display the preprint in perpetuity. It is made available under aCC-BY 4.0 International license.

Supplementary Materials

Live imaging datasets

All zebrafish experiments were performed according to the Swiss Law and the Kantonales Veterinäramt of Kanton Basel-Stadt (licenses #1035H). The FISH1 dataset was produced in the Schier lab in the University of Basel (*9*). The dataset recorded the early stage of the development process of a zebrafish embryo. Staging of the embryos was performed according to (*49*), available at the Zebrafish Information Network (ZFIN) (*73*). There are more than one million cells in 192 time points. We used our method to generate a preliminary tracking result first, and then a part of the tracks with biological interests were selected and professionally curated twice. As a result, we generated 37 lineages containing more than 32 thousand associations in total.

The FISH2 dataset is from Keller lab in HHMI Janelia Research Campus, and has been demonstrated in (*39*). It recorded a zebrafish embryo as well but in a relatively longer time duration. We selected 100 time points (300-399) at the middle stage as a complement of FISH1, which had the same amount of cells. We also manually curated based on our results and selected 36 lineages as the ground truth.

The MOUSE dataset is from Keller lab in HHMI Janelia Research Campus recording the development of mouse embryo. Besides, it serves as the evaluation dataset in Linajea (*41*, *42*). We selected the middle stage with 50 frames (250-299) for evaluation, which offers a moderate level of difficulty. Due to occasional errors and ambiguities in the annotations, we combined publicly available annotations with our own corrections and annotations to create the final evaluation ground truth.

The DRO dataset is from Hufnagel group in EMBL Heidelberg (*74*) recording the development of *Drosophila melanogaster* embryo, and has been partly annotated and tested in (*75*). The annotation is claimed as a combination of automatic segmentation with refinement and manual association. However, we noticed the existing of mistakes, such as broken tracks. We manually checked some annotations and selected 62 correct lineages as the ground truth to ensure reliable evaluation.

Peer methods

The first one is Tracking with Gaussian Mixture Models (TGMM) (*1*, *39*), which is an unsupervised

bioRxiv preprint doi: https://doi.org/10.64898/2026.03.12.711203; this version posted March 16, 2026. The copyright holder for this preprint (which was not certified by peer review) is the author/funder, who has granted bioRxiv a license to display the preprint in perpetuity. It is made available under aCC-BY 4.0 International license.

algorithm that uses watershed (*76*) to generate segmentation candidates, and then uses 3D Gaussian mixture model to associate cells in two adjacent time points greedily and determine the final segmentation, where results in every time point are also the prior for the next time point.

The second one is Linajea (*41*, *42*), a two-stage algorithm whose detection is based on 3D U-Net (*77*). The tracking stage considers all cell associations in the whole data including divisions, formulates them as an integer linear programming (ILP) problem, and can obtain a global optimum theoretically. It is unavoidable to bring NP-hardness as mentioned before, while the time and memory consumptions are unacceptable for large-scale data. As a result, Linajea processes real data block-wise and is content with an approximate solution to the original optimization problem.

The third one is Ultrack (*38, 43*), which handles the detection and tracking task jointly. It uses Ultrametric Contour Maps (UCM) (*78*) to generate segmentation candidates hierarchically, and then formulates the tracking problem into ILP similar to Linajea. Ultrack does not determine the segmentation results at the beginning. Instead, it selects the optimal segmentation results from hierarchies while solving for the tracking results.

Evaluation metrics

Due to the sparse labeling of cells in ground truth, we define that a detected cell is successfully recognized if it is the closest one to a cell in the ground truth, and the distance is smaller than the cell radius (Fig.S1A). A detected cell can be matched to at most one ground truth cell and vice versa.

Detection FP/FN: If the centroids in the densely annotated ground truth are less than a certain distance from the centroids in the detection results, we match them and calculate FP/FN based on this (Fig.S1B).

Euclidean distance metric: For each annotated centroid, we determine which nucleus it was assigned to in the automatic reconstruction. Then, we measure the Euclidean distance between the annotated centroid and the centroid of the nucleus from the automatic reconstruction. If the detection results have severe under segmentation, this metric will be biased higher (Fig.S1C).

Nearest neighbor normalized distance metric: For each annotated data point, for which we have calculated the Euclidean distance metric explained above, we normalize the distance measure by the distance to the nearest neighbor in the automatic reconstruction. If there is severe over segmentation in the detection results, the normalized metric will be biased towards a larger value (Fig.S1D).

Linkage TP/FP/FN: For each cross-frame association, an association is regarded as a true positive (TP) if both the parent and daughter cells are successfully recognized and match an association in the ground truth. If at least one cell is recognized but the association is wrong, it is treated as a false positive (FP). If an association in the ground truth cannot find any matched cell or if the matched cells don't have a track, the association is miss detected and treated as a false negative (FN) (Fig.S1E). Since cell division is a process that occurs over a period of time, we adopt a lenient evaluation strategy for divisions that occur earlier or later than in the ground truth, meaning they are not counted as FP or FN errors (Fig.S1F).

Errors per ground truth edge: We define FP/FN/Sum per ground truth edge as the ratio of FP/FN/(FP + FN) to the number of edges in the ground truth, respectively. These three metrics can quantitatively evaluate the performance of tracking.

Average error-free length: We use the complete lineage annotated from beginning to end as the unit,

bioRxiv preprint doi: https://doi.org/10.64898/2026.03.12.711203; this version posted March 16, 2026. The copyright holder for this preprint (which was not certified by peer review) is the author/funder, who has granted bioRxiv a license to display the preprint in perpetuity. It is made available under aCC-BY 4.0 International license.

average error-free length is the number of consecutive time points over which we encountered no tracking errors. Due to imaging defects, some lineages in the ground truth are incomplete, so we weight them by track length (Fig.S1G).

Proportion of error-free tracks: This metric represents the proportion of completely correct lineages to the total annotated lineages (Fig.S1G).

Application scenarios

Low signal-to-noise ratio: ITEC accurately models the noise and has several feedback ideas to iteratively improve tracking accuracy. Therefore, it has an absolute advantage in processing low signal-to-noise ratio data, which is difficult to achieve based on deep learning methods.

Large scale data: In segmentation module, ITEC adopt a parallel strategy to accelerate seed growth and boundary generation. In tracking module, it applies the most advanced machine learning techniques for data association, and despite multiple iterations, it can still achieve high running speed.

High accuracy requirement: ITEC achieves high tracking accuracy through effective error correction module design. This is crucial in the reconstruction of the entire embryonic developmental lineages. ITEC can almost achieve zero error rate in the parts with clearer imaging, ensuring the integrity and accuracy of the lineages.

Multi application scenarios: ITEC has been applied in various embryos such as zebrafish, mouse, *Drosophila*, etc., achieving the highest performance. Meanwhile, it can handle both local regions of interest and the entire embryonic development process, with a wide range of application scenarios and potential.

User-friendly design

ITEC strives to be user-friendly, primarily manifested in three aspects. First, it is fully based on unsupervised machine learning techniques, which eliminates the need for data annotation. Users only need to adjust parameters that have practical biological significance or are intuitively adjustable (Table S3). For example, we set thresholds with clear meanings for grayscale values, such as *background intensity*, *intensity upper bound*, and *intensity lower bound*. Users can easily obtain these parameters using mainstream image processing software available today. Additionally, there are parameters for balancing false positives (FP) and false negatives (FN), including *curvature threshold* and *division threshold*, which allow users to control the number of detected cells and confidence level according to their specific needs.

Second, we simultaneously design a UI interface and provide a parameter table, enabling users to conveniently adjust parameters and run the pipeline across different devices. Meanwhile, we have integrated the tracking results with Mastodon (*47*), a mainstream large-scale embryonic cell tracking Fiji plugin (*48*). This allows users to easily use the platform to view tracking results, lineage trees, and other information, as well as curate and analyze the results.

Third, we provide users with a comprehensive user manual, which includes multiple example parameter tables tested during our development process. This facilitates quick user onboarding and enables users to rapidly adjust parameters in practical applications.

Distribution dispersion measurement

bioRxiv preprint doi: https://doi.org/10.64898/2026.03.12.711203; this version posted March 16, 2026. The copyright holder for this preprint (which was not certified by peer review) is the author/funder, who has granted bioRxiv a license to display the preprint in perpetuity. It is made available under aCC-BY 4.0 International license.

The mean distance dispersion index reflects the overall dispersion degree by calculating the average distance between all pairs of points. A higher value of this index indicates a more dispersed cell distribution. This index can well take the contribution of all cells into account and avoid being affected by outlier points. We define the coordinates of the points as x_i (i = 1, 2, …, n), and their average distance dispersion index is:

$$MPD(\boldsymbol{X}) = \frac{2}{n(n-1)}\sum_{i=1}^{n}\sum_{j=i+1}^{n}\|\boldsymbol{x}_i - \boldsymbol{x}_j\|$$

Velocity map and variance map calculation

At the TM230 of FISH2 (corresponding to 75% epiboly), the velocity of each cell was derived from the cell tracking results of the 20 preceding and subsequent frames. Subsequently, the velocity of each cell position was obtained by averaging the velocities of the 10 neighboring cells around that cell. The variance of each cell is calculated from the velocity variances of up to 50 cells within a certain range around that cell.

Online methods

Step 1: Pre-processing

Deconvolution

In general, the output of an optical imaging system can be described as a convolution between the input signal and the point spread function (PSF) with additive noise $O = h * I + N$, or in the frequency domain,

$$\mathcal{F}(O) = \mathcal{F}(h)\mathcal{F}(I) + \mathcal{F}(N), \tag{1}$$

where I, O, N are the input time-series signals, output videos, and noise. h is the time-invariant PSF and F is the Fourier transform. Especially for fluorescence microscopy, deconvolution is crucial to restore the real signal I and can help to avoid under-segmentation. We formulate the deconvolution problem as a least squares estimation when h is given:

$$min\|\mathcal{F}(O) - \mathcal{F}(h)\mathcal{F}(I)\|^2 \tag{2}$$

Rather than deriving the solution $\mathcal{F}(I) = [\mathcal{F}(h) * \mathcal{F}(h)]\mathcal{F}(h) * \mathcal{F}(O)$ directly, to solve the ill-posed problems and suppress the noise, we use the Landweber algorithm (*79*) to estimate $\mathcal{F}(I)$ iteratively:

$$\nabla\mathcal{F}(I) = -\mathcal{F}(h) * \|\mathcal{F}(O) - \mathcal{F}(h)\mathcal{F}(I)\| \tag{3}$$

$$\mathcal{F}(I)^{k+1} = \mathcal{F}(I)^k - \lambda\nabla\mathcal{F}(I)^k$$
$$= \mathcal{F}(I)^k - \lambda\mathcal{F}(h) * \mathcal{F}(h)\mathcal{F}(I)^k + \lambda\mathcal{F}(h) * \mathcal{F}(O) \tag{4}$$

where λ is the step size and set to 0.9 in all experiments. If h is unknown, as a rule of thumb, an appropriate Gaussian kernel along the z-axis performs better than blind deconvolution approaches.

Rigid registration

Similar to most cell tracking methods, ITEC assumes the cell motions are small, which may not

bioRxiv preprint doi: https://doi.org/10.64898/2026.03.12.711203; this version posted March 16, 2026. The copyright holder for this preprint (which was not certified by peer review) is the author/funder, who has granted bioRxiv a license to display the preprint in perpetuity. It is made available under aCC-BY 4.0 International license.

always be true for real data. Cell motions can be composed of three components: global motion mainly because of the whole embryo motion and rotation during experiments, tissue-level motion because of the development, and cell-level nearly Brownian motion. To eliminate the influence of global motion, we get the rigid transformation between adjacent time points $I(t)$ and $I(t+1)$ by mean squared error minimization:

$$\min \left\| \begin{bmatrix} \boldsymbol{I(t+1)} \\ \mathbf{1} \end{bmatrix} - \begin{bmatrix} \boldsymbol{R} & \boldsymbol{v} \\ \mathbf{0} & \mathbf{1} \end{bmatrix} \begin{bmatrix} \boldsymbol{I(t)} \\ \mathbf{1} \end{bmatrix} \right\|^{\mathbf{2}}, \tag{5}$$

where R and v represent the rotation matrix and motion vector. It is worth mentioning that we do not transform the image data but only record the transformation matrix. Transformed data will consume much larger storage but lose more information than the original data. Rigid registration is also applied to stitch different angles of the same time point, which will be discussed in the post-processing.

Motion flow estimation

Rigid registration can make most cells satisfy the small motion assumption, while exceptions still exist. We treat these motions as tissue-level and applied patch-wise and pyramidal Lucas-Kanade optical flow (*45*) to roughly estimate these motions and register images non-rigidly.

Optical flow is a broadly applied motion estimation approach. However, the dense optical flow has quite expensive computation and storage overhead while the sparse optical flow is not applicable since cell locations may change during the error correction stage. As a compromise, we apply patch-wise and pyramidal Lucas-Kanade optical flow to roughly capture cell motion and register images non-rigidly. The basic idea is to downsample images and roughly estimate a global motion as initialization, and then upsample images, divide them as patches, and estimate the motion of every patch independently. The upsampling and patch-wise motion estimation can be executed multiple times while maintaining the same patch size but a smaller field of view for a patch.

To start with, we introduce the method of rigid registration through optical flow under the following assumptions: (i) The cell motion is small. (ii) The cell intensity does not change too much in two images. (iii) Adjacent cells share similar motions. The small motion assumption will be satisfied when applying pyramidal optical flow, and the other two assumptions are naturally satisfied by embryonic imaging data. Based on the assumptions, for a pixel at location (x, y, z, t) in a video I, it should have the same intensity at the next time point:

$$I(x+\delta x, y+\delta y, z+\delta z, t+\delta t) = I(x, y, z, t) \tag{6}$$

where $(\delta x, \delta y, \delta z)$ is the motion distance along coordinate axes. Because of the small motion assumption, we take the first-order approximation:

$$I(x, y, z, t) + \frac{\partial I}{\partial x}\delta x + \frac{\partial I}{\partial y}\delta y + \frac{\partial I}{\partial z}\delta z + \frac{\partial I}{\partial t}\delta t = I(x, y, z, t) \tag{7}$$

$$\frac{\partial I}{\partial x}\frac{\partial x}{\partial t} + \frac{\partial I}{\partial y}\frac{\partial y}{\partial t} + \frac{\partial I}{\partial z}\frac{\partial z}{\partial t} + \frac{\partial I}{\partial t} = 0 \tag{8}$$

$\frac{\partial I}{\partial x}, \frac{\partial I}{\partial y}, \frac{\partial I}{\partial z}$ are image intensity gradients along different directions, and $(\frac{\delta x}{\delta t}, \frac{\delta y}{\delta t}, \frac{\delta z}{\delta t})$ is the cell

bioRxiv preprint doi: https://doi.org/10.64898/2026.03.12.711203; this version posted March 16, 2026. The copyright holder for this preprint (which was not certified by peer review) is the author/funder, who has granted bioRxiv a license to display the preprint in perpetuity. It is made available under aCC-BY 4.0 International license.

motion velocity waiting for estimation. To robustly estimate the motion velocity, we consider n multiple pixels (x_1, y_1, z_1, t), (x_2, y_2, z_2, t), …, (x_n, y_n, z_n, t) in one image or a patch. Notably, considering all pixels may not bring better estimation since some background pixels are pure noise. Then we have

$$\begin{bmatrix} \frac{\partial I_1}{\partial x} & \frac{\partial I_1}{\partial y} & \frac{\partial I_1}{\partial z} \\ \frac{\partial I_2}{\partial x} & \frac{\partial I_2}{\partial y} & \frac{\partial I_2}{\partial z} \\ \vdots & \vdots & \vdots \\ \frac{\partial I_n}{\partial x} & \frac{\partial I_n}{\partial y} & \frac{\partial I_n}{\partial z} \end{bmatrix} \begin{bmatrix} \frac{\delta x}{\delta t} \\ \frac{\delta y}{\delta t} \\ \frac{\delta z}{\delta t} \end{bmatrix} = -\begin{bmatrix} \frac{\partial I_1}{\partial t} \\ \frac{\partial I_2}{\partial t} \\ \vdots \\ \frac{\partial I_n}{\partial t} \end{bmatrix} \tag{9}$$

or in short,

$$\mathbf{Av} = -\mathbf{I}_t \tag{10}$$

Then $v = \left[\frac{\delta x}{\delta t} \frac{\delta y}{\delta t} \frac{\delta z}{\delta t}\right]^T$ can be solved by least squares with the given objective function:

$$\min ||\boldsymbol{I_t} + \boldsymbol{Av}||^2 \tag{11}$$

The optimum $\mathbf{v}^* = -(\mathbf{A}^T\mathbf{A})^{-1}\mathbf{A}^T\mathbf{I}_t$ is the estimated velocity. Then we upsample the image, divide the image as patches, and refine the velocity estimation with x^* as an initialization. The following estimations are exactly the same as the first one if the patches are processed independently. However, some patches may be at the embryo boundary, contain very few foreground pixels, and cannot be correctly estimated without the help of neighbors. It is natural to assume adjacent patches should have similar motions and consider the similarities when estimation. We assume that there are m patches, describe the patch relationship as a graph, and use the mean squared error to describe the similarity cost:

$$\sum_{(i,j)\in\mathcal{G}} \left\|\mathbf{v}_i - \mathbf{v}_j\right\|^2 \tag{12}$$

Finally, the objective function is the least squares estimation of patch motions with the regularization of similarities:

$$\left\|\begin{bmatrix} \mathbf{I}_{t1} \\ \mathbf{I}_{t2} \\ \vdots \\ \mathbf{I}_{tm} \end{bmatrix} + \begin{bmatrix} \mathbf{A}_1 & \mathbf{0} & \dots & \mathbf{0} \\ \mathbf{0} & \mathbf{A}_2 & \dots & \mathbf{0} \\ \vdots & \vdots & \ddots & \vdots \\ \mathbf{0} & \mathbf{0} & \dots & \mathbf{A}_m \end{bmatrix} \begin{bmatrix} \mathbf{v}_1 \\ \mathbf{v}_2 \\ \vdots \\ \mathbf{v}_m \end{bmatrix}\right\|^2 + \beta \sum_{(i,j)\in\mathcal{G}} \left\|\mathbf{v}_i - \mathbf{v}_j\right\|^2 \tag{13}$$

where β a regularization factor. In general, we prefer to set β to 1~5% of the mean intensity, which can contribute but not dominate the estimation. The optimal solution v_i^* will be treated as the motion velocity of the center of patch i. Therefore, for any location in the image, we can estimate its velocity through the interpolation of adjacent patch centers.

Step 2: Initial Detection

Accurately identifying cells within complex, crowded populations and performing pixel-level segmentation presents significant challenges. On one hand, strict differentiation between noise and

bioRxiv preprint doi: https://doi.org/10.64898/2026.03.12.711203; this version posted March 16, 2026. The copyright holder for this preprint (which was not certified by peer review) is the author/funder, who has granted bioRxiv a license to display the preprint in perpetuity. It is made available under aCC-BY 4.0 International license.

genuine signals is required. Without precise noise modeling, many noise artifacts can be misclassified as cells. On the other hand, intrinsic imaging heterogeneity leads to intercellular variations. While most cells appear bright in the center and dark at the periphery, some exhibit internal textures resembling multiple cells, potentially causing over-segmentation or under-segmentation. To address these issues, first, we perform robust estimation of background noise by assuming noise follows an independent Gaussian distribution and estimating its variance to establish a foundation for subsequent steps. Second, because the maximum principal curvature (short for principal curvature or curvature in this section) encapsulates information about cell boundaries and intercellular gaps, we compute it and integrate results from the first step to accurately model and quantify the curvature distribution of noise (*46*). This separates cells from the background, excludes clearly non-cellular regions, and provides coarse positional information. Third, we utilize curvature information to grow cell boundaries, achieving precise pixel-level segmentation for enhanced downstream tracking (Fig. S4).

Variance estimation

To model background noise, we first require accurate estimation of its variance. We employ a robust variance estimation method assuming independent Gaussian noise in the background. This involves applying small-scale filtering to the image, then estimating the true variance using the distribution of intensity differences between each pixel and its neighbors, with correction coefficients generated via simulation. This approach leverages local information to mitigate global intensity variations under unknown signal distributions. The operation is performed on each z-slice of the 3D image, with the median variance across z-slices serving as the final estimate.

Boundary map generation

Principal curvature provides essential characterization of cell boundaries, particularly in dense populations, where it prevents over-merging and improves segmentation accuracy. However, curvature is sensitive to intracellular noise and struggles to distinguish internal textures from noise, making it necessary to perform smoothing. With small smoothing parameters, cell contours are preserved clearly without distortion, but cells with gaps may be over-segmented, making this suitable for boundary extraction. With large smoothing parameters, intracellular regions become smoother and more homogeneous, making this suitable for cell identification.

To fully leverage this information, we implement a multi-scale principal curvature (MSPC) method based on order statistics through computing the normalized principal curvature with specific smoothing factors derived from the maximum eigenvalue of the modified Hessian matrix.

$$H(i) = \begin{bmatrix} \frac{I_{xx}(i)}{S_{xx}} & \frac{I_{xy}(i)}{N_{xy}} & \frac{I_{xz}(i)}{N_{xz}} \\ \frac{I_{yx}(i)}{N_{xy}} & \frac{I_{yy}(i)}{S_{yy}} & \frac{I_{yz}(i)}{N_{yz}} \\ \frac{I_{zx}(i)}{N_{xz}} & \frac{I_{zy}(i)}{N_{yz}} & \frac{I_{zz}(i)}{S_{zz}} \end{bmatrix} \tag{14}$$

where I_{xx}, I_{xy}, I_{xz},... are the second-order partial derivatives of the smoothed image evaluated at the pixel i; S_{xx}, S_{yy}, S_{zz} is the sum of all pixel's squared value in the second-order derivative of the Gaussian smooth kernel along the specific direction; The N_{xy} is the $\sqrt{S_{xx}S_{yy}}$.

bioRxiv preprint doi: https://doi.org/10.64898/2026.03.12.711203; this version posted March 16, 2026. The copyright holder for this preprint (which was not certified by peer review) is the author/funder, who has granted bioRxiv a license to display the preprint in perpetuity. It is made available under aCC-BY 4.0 International license.

We introduce two key enhancements. First, due to the z-direction characteristics of our data, we compute both 3D curvature and 2D curvature for each z-slice. Second, using the previously estimated background variance, we improve the curvature normalization across filtering scales. For 2D and 3D, background curvature follows a Gaussian distribution, so we normalize curvature at all scales to a standard normal distribution. Our current objective is to select cellular core regions. Based on accurate noise modeling and after excluding implausible filtering parameters, we use the minimum principal curvature across all scales to indicate seed information, applying a tunable threshold (typically -5) where values below this denote seeds. Simultaneously, we use the maximum principal curvature across all scales to indicate boundary information, where values above +3 are classified as boundaries and excluded from seed regions. This approach fully leverages the characteristics of principal curvature across different filtering scales, yielding more robust and accurate results.

Min-cut seed growth

The boundary growth is used to grow the seeds obtained in the previous section to obtain accurate cell boundaries. In fact, the growth of cell boundaries is to find the continuous pixels around the seed with the maximum sum of principal curvatures. Therefore, we model the problem as a minimum cut optimization problem with one source and multiple sinks.

For a specific seed *n*, we define the pixels belonging to the cell region grown from it as R_n, and all other pixels as $\overline{R_n}$. The boundary between these two groups can be represented using pairs of pixels, C_n. Finding the set of boundary pairs C_n that minimizes the sum of $G(i)$ and $G(j)$ across all pairs:

$$\underset{C_n}{argmin} \sum_{(i,j)\in C_n} (G(i) + G(j)) \tag{15}$$

Here, $G = \frac{1}{max(G,T)^2}$, where G is the map of principal curvature for each pixel, and T is a minimum capacity threshold introduced to enhance robustness. It is worth noting that we combined the curvature of 3D and 2D to calculate c_{ij}, in order to compensate for the erroneous growth caused by missing *z*-direction information. In this way, the problem of finding the boundary pixels with the maximum value in the curvature map G is transformed into finding the minimum values within G. This objective function also indirectly minimizes the boundary length.

To efficiently solve this problem, the problem is reformulated as a minimum cut problem. As shown in Fig. S4, the optimization graph constructed for Seed 1 includes all foreground pixels, with each node i representing a pixel. An edge e_{ij} connects every pair of neighboring nodes, and its capacity is defined as $weight(e_{ij}) = G(i) + G(j)$. All pixels corresponding to the seed are considered the source, while the background and other seeds are considered the sink. With this graph design, the minimum cut between the source and sink is calculated. This minimum cut represents the cell boundary, and the pixels enclosed by it are labeled as the detection result for the corresponding seed.

Step 3: Tracking

Problem statement

The problem of object tracking can be formulated as an MAP problem. Here we discuss the most widely used formulations that consider only one-to-one matchings (*80*).

bioRxiv preprint doi: https://doi.org/10.64898/2026.03.12.711203; this version posted March 16, 2026. The copyright holder for this preprint (which was not certified by peer review) is the author/funder, who has granted bioRxiv a license to display the preprint in perpetuity. It is made available under aCC-BY 4.0 International license.

Let $\mathcal{X} = \{x_i\}$ be a set of detections, where x_i is a vector containing the position, appearance, and time index of detection i. A track $T_k = \{x_{k_1}, \dots, x_{k_n}\}$, $x_{k_i} \in \mathcal{X}$ is a ordered list of detections and

$$T_k \cap T_l = \emptyset, \forall k \neq l \tag{16}$$

An association hypothesis is a set of non-overlap tracks $\mathcal{T} = \{T_k\}$. Note that the $\mathcal{T}$ may not cover all the detections of $\mathcal{X}$ as detections can be false positive. The purpose of identity inference is to find the hypothesis $\mathcal{T}$ with the highest posterior probability:

$$\begin{aligned} \mathcal{T}^* &= \arg\max_{\mathcal{T}} P(\mathcal{T}|\mathcal{X}) \\ &= \arg\max_{\mathcal{T}} P(\mathcal{X}|\mathcal{T})P(\mathcal{T}) \\ &= \arg\max_{\mathcal{T}} \prod_i P(x_i|\mathcal{T}) \prod_k P(T_k). \end{aligned} \tag{17}$$

assuming conditional independence of detections given the hypothesis $\mathcal{T}$ and independence between tracks, i.e. objects move independently.

Assume each $P(x_i|\mathcal{T})$ follows a unique Bernoulli distribution $B(1, \theta_i)$, with a preset parameter θ_i indicating the probability that x_i is mistakenly detected and thus should be excluded in the tracks:

$$P(x_i|\mathcal{T}) = \begin{cases} 1 - \theta_i, & \exists T_k \in \mathcal{T}, x_i \in T_k \\ \theta_i, & \text{otherwise} \end{cases} \tag{18}$$

Since we only consider unary and pairwise relationships between detections, a track $T_k = \{x_{k_1}, \dots, x_{k_n}\}$ can be modeled as a Markov chain whose probability is

$$P(T_k) = P_{enter}(x_{k_1}) \prod_{i=1}^{n-1} P(x_{k_{i+1}}|x_{k_i}) P_{exit}(x_{k_n}) \tag{19}$$

$P_{enter}(x_{k_1})$ is the probability that x_{k_1} is the initial point of track T_k. Similarly, $P_{exit}(x_{k_n})$ is the probability that x_{k_n} is the terminate point of track T_k. By taking the negative logarithm of all the probabilities, and remapping $\mathcal{T}$ into indicators $f_i, f_i^{en}, f_i^{ex}, f_{i,j}$, the MAP problem can be converted to:

$$\begin{aligned} f^* &= \arg\min_{\mathcal{T}} \Big(-\sum_{\mathrm{i}} \log P(x_i|\mathcal{T}) - \sum_{\mathrm{k}} \log P(T_k)\Big) \\ &= \arg\min_f \Big(\sum_i C_i f_i + \Big(\sum_i C_i^{en} f_i^{en} + \sum_{i \neq j} C_{i,j} f_{i,j} + \sum_i C_i^{ex} f_i^{ex}\Big)\Big) \\ &\text{s.t.} \quad f_i, f_i^{en}, f_i^{ex}, f_{i,j} \in \{0,1\} \\ &\text{and} \quad f_i^{en} + \sum_j f_{j,i} = f_i = f_i^{ex} + \sum_j f_{i,j} \end{aligned} \tag{20}$$

with

$$C_i^{en} = -\log P_{enter}(x_i), \quad C_i^{ex} = -\log P_{exit}(x_i)$$

bioRxiv preprint doi: https://doi.org/10.64898/2026.03.12.711203; this version posted March 16, 2026. The copyright holder for this preprint (which was not certified by peer review) is the author/funder, who has granted bioRxiv a license to display the preprint in perpetuity. It is made available under aCC-BY 4.0 International license.

$$C_{i,j} = -\log P\left(x_i \middle| x_j\right), \quad C_i = \log \frac{\theta_i}{1-\theta_i} \tag{21}$$

Here $f_i = 1$ indicates that detection x_i is included in a track of $\mathcal{T}$ and $f_i = 0$ otherwise. $P(x_i|\mathcal{T})$ can be rewritten as $(1-\theta_i)^{f_i}\theta_i^{(1-f_i)}$. $f_i^{en} = 1$ or $f_i^{ex} = 1$ indicates that x_i is the initial or terminate point of a track in $\mathcal{T}$. $f_{i,j} = 1$ means that detection x_i is followed by detection x_j in the same track in $\mathcal{T}$.

Constraints in Equation 20 indicate that each detection can participate in at most one track and there is no splitting or merging of any track. We can see $f_i = 1$ forces detection x_i to be incident with at most one previous detection and at most one following detection in a track, so x_i will participate one and only one track. On the other hand, $f_i = 0$ automatically rules out the detection x_i from participating in any track, where f_i^{en}, $f_{\_,i}$, f_i^{ex}, and $f_{i,\_}$ will all be zeros.

Calculate affinity score between cells

A key component for a good tracker is the affinity score measuring the similarity among detections. However, for objects with a flat appearance like cells or particles, discriminative features are hard to design. From our observation, the motion and morphological changes of cells are relatively slow across time. Thus, we proposed to design an affinity score on the basis of the following assumption.

Assumption 1. Reasonably high temporal resolution of imaging. The temporal resolution is high enough such that, between any two consecutive frames, a cell in frame *t*+1 is the spatially closest one to itself in frame *t* among all cells in frame *t*+1.

Such an assumption is inevitable considering the lack of texture and morphology differences among cells. Based on this assumption, the most effective and faithful affinity score design is to measure the morphological similarity like the territory overlapping ratio between two detections.

However, either overlapping ratio or center distance suffers from the ellipsoid shape of cells. As is shown in Fig. S6, the two detections in (a) have the same overlapping ratio with those in (c), while has the same center distance as those in (b). However, from our point of view, the cells in (a) have smaller morphological changes than (c), but the evidence is not as strong the those in (b). Thus the desired score be able to differentiate these three conditions. As a single standard cannot achieve this task, we proposed the new design jointly consider these two criteria.

Let's use D_i and D_j to denote the two adjacent detections as is shown in Fig. S6. $p \in D_i$ represents a pixel p located inside D_i. For two pixels $p \in D_i$ and $q \in D_j$, we define their distance $d(p,q)$ as their Euclidean distance. We first define the distance from a pixel p to a detection D_j as

$$d\left(p, D_j\right) = \min_{q \in D_j} d\,(p,q) \tag{22}$$

Then we can define the distance from detection D_i to D_j as

$$d\left(D_i, D_j\right) = \frac{1}{n_i} \sum_{p \in D_i} d\left(p, D_j\right), \tag{23}$$

where n_i is the number of pixels in D_i. With the score in Equation 23, if detection D_i has larger

bioRxiv preprint doi: https://doi.org/10.64898/2026.03.12.711203; this version posted March 16, 2026. The copyright holder for this preprint (which was not certified by peer review) is the author/funder, who has granted bioRxiv a license to display the preprint in perpetuity. It is made available under aCC-BY 4.0 International license.

ratio of pixels overlapped with D_j, these pixels will contribute zero to the cumulative distance, but weight down the average distance. Thus the two conditions in Fig. S6A and Fig. S6B will have different distances. For Fig. S6A and Fig. S6C, the non-overlapping pixels in D_i in Fig. S6A clearly has smaller average distance to D_j than those in Fig. S6C and thus can also be differentiated. The distance in Equation 23 is directional, which means $d(D_i, D_j) \neq d(D_j, D_i)$ holds for most of the cases. If we want to have a consistent non-directional distance between two detections D_i and D_j, we can define it as

$$\omega(D_i, D_j) = \max\left(d(D_i, D_j), d(D_j, D_i)\right) \tag{24}$$

The distance function ω in Equation 24 can also discriminate the condition that a small detection is fully overlapped within a large detection. The directional distance from the small detection to the large one will be zeros, but if we measure it using the counter direction, it will be large.

Minimum-cost circulation framework

Current minimum-cost flow solvers are not efficient enough on large-scale datasets. These solvers utilize the strategy that pushes successive shortest paths until optimum (*27*) or binary searches the optimum given pre-determined flow amounts. For the scenario of cell tracking, the best time complexity is $O(\min\left(n^{\frac{2}{3}}, m^{\frac{1}{2}}\right)\log^2(n))$ (*81*), where *n* and *m* are the numbers of cells and potential links, and the best real performance is to take several hours to track millions of cells. To solve the MAP problem mentioned above efficiently, we use CINDA (CIrculation Network-based Data Association) to solve the problem (*44*) (Fig. S5). This framework reforms the MAP problem as a minimum-cost circulation problem and maintains the same optimal solution. It has not only a superior $O\left(n^{\frac{1}{2}}m\log(n)\right)$ theoretical guarantee but also a tenfold practical efficiency improvement, which only takes minutes to track millions of cells and provides the feasibility of our iterative tracking strategy.

Step 4: Error Correction

Cell under-segmentation and over-segmentation

Due to the ambiguity between adjacent cells or the intrinsic signal heterogeneity inside a cell, we may encounter segmentation errors, which mainly include two types. The cell under-segmentation indicates that there are indeed two cells (or more cells) contained in one detection. We wrongly merged the two cells. Cell over-segmentation is just the opposite, in which we wrongly split one cell into two (or more) detections. These two types of errors result in the same consequence, which is that two detections will have the same spatially closest neighbor in the adjacent frame. These errors have a clear difference from the normal cell division. For cell division, the two resultant cells will show clear spatial dis-connectivity, while for the segmentation errors, the two detections are tightly connected with each other. In our iterative framework, these segmentation errors are processed by module one.

We first designed a new network based on minimum-cost circulation, which links detections across time while allowing detections to split or merge. As is shown in Fig. 3D, a cell is mistakenly split as two detections in frame *t*+1, while the segmentation is correct in frame *t* and *t*+2. We add an arc

bioRxiv preprint doi: https://doi.org/10.64898/2026.03.12.711203; this version posted March 16, 2026. The copyright holder for this preprint (which was not certified by peer review) is the author/funder, who has granted bioRxiv a license to display the preprint in perpetuity. It is made available under aCC-BY 4.0 International license.

from s to h_1 the post-node of detection 1 and thus h_1 can have at most two units of input and output flow. By the same token, we add one arc from o_4 , the pre-node of detection 4 to s, which allows at most two units of flow going through it. Now detection 2 and 3 can both be linked to detection 1 and also can both link to detection 4. The over-merged cells can also be processed with this design where the arcs are appended to the detection consists of two cells.

It is worthy to note that, we usually cannot determine a two-to-one linking is cell over-segmentation or under-segmentation directly from these two or three adjacent frames as evidence is still limited. Otherwise, we can directly force the over-split detections to be merged or an over-merged detection to be split. However, utilizing our new network design, the over-split or over-merged detections will be involved in a long trace covering dozens of frames. With the rich spatial-temporal relationships inferred from this trace, we can resolve the errors by re-merging and re-splitting the ambiguous detections.

We designed four criteria to determine the one-to-two and two-to-one linking is over-segmentation or under-segmentation. In practice, the four criteria will be applied sequentially as the confidence of the conclusions drawn from these them are different.

Voting from adjacent frames. The first criteria is to utilize the information from adjacent frames. For example, for the detection 1274 in the trace in Fig. S7A, the former three frames and the latter three frames all contain two detections. If we view the condition in one frame as a Bernoulli trial with over-segmentation probability 0.5. The probability of 6 out of 7 frames are over-split is less than 5%. Thus, we conclude that detection 1274 should be forced to split (Fig. S7B). Similarly, we can tell detection 3696 and 3697 should be forced to merge.

Voting from disconnected detections. The second criterion is based on the assumption that if a cell is over-split at some time point, the resultant two detections should be close and touched. Thus disconnected detections can be viewed as a piece of evidence indicating that there are two cells intertwined in this track. For example, for the track in Fig. S7A, the detection 55 and 78 are in the same frame but totally disconnected (Fig. S7B). Based on our assumption, we can conclude that the two-to-one linking temporally closest to the disconnected detections should be forced to split, which means the detection 1274 should be split. Note that, this splitting decision for detection 1274 has been made using our first criterion. In practice, the second criterion will only be applied if the first criterion failed to give a solid conclusion.

Voting from detections with similar size. We will conduct the third criterion if the former two both fail. The third criterion is based on the assumption that the territory size of a cell should be relatively consistent. For example, from $t = 19$ to $t = 40$, there is only one cell involved in the track with a relatively similar territory size. Among these 22 frames, we have two detections in 5 frames, while one detection in all other frames. With the same idea from our first criterion, the probability of 17 out of 22 frames are over-merged is less than 5%. Thus, we conclude that detections in the 5 frames are all over-split and should be forced to merge.

Try splitting. If all the previous criteria fail, we will try splitting the detection based on the gaps detected by principal curvature. If the resultant two detections have a high affinity score with the two detections in the former or latter frame, we will keep them. For example, for the detection 1742 in Fig. S7B (t=15), if we split it, the resultant two detections will have high morphological similarity

bioRxiv preprint doi: https://doi.org/10.64898/2026.03.12.711203; this version posted March 16, 2026. The copyright holder for this preprint (which was not certified by peer review) is the author/funder, who has granted bioRxiv a license to display the preprint in perpetuity. It is made available under aCC-BY 4.0 International license.

with detection 1622 and 1623. Thus, we will choose to force detection 1742 to split. The reason we did this is to retrieve the false negative gaps when we conduct cell detection and segmentation. The gaps used here must be insignificant and missed in gap testing, otherwise we should have already split the corresponding detection.

With these four criteria, majority of the under-segmentation and over-segmentation cells should be corrected, the rest of which will be left for the next iteration when more evidence is accumulated. With such an iterative framework, we indeed are not afraid of making wrong decisions on the splitting and merging. As long as majority decisions are correct, the wrong decisions conducted will highly likely be corrected in the next iterations.

Cell missing

Fig. 3B shows a typical example of cell missing. Here we call the detection in the former frame as a parent of the missed detection and the one in the latter frame as a kid of the missed detection. The cell in frame t is missed while its parent and kid are both detected. Thankfully, our minimum-cost circulation-based data association framework has the ability to deal with this kind of cell missing.

Fig. 3C illustrates how it works. As is shown in Fig. 3B, there are two cells in the field of view indicated by different colors. The blue cell forms a track that consists of detection 2, 3 and 5. The orange cell has only two detections since its footprint in frame t+1 was missed. To continue the tracking of the orange cell, our network shown in Fig. 3C allows the linkages between detections beyond adjacent frames. Thus, though detection 1 has no appropriate candidate to link in frame t, it can jump over this frame and directly link to detection 4. In practice, we can adjust the number of frames that are allowed for jumping by a rough estimation of the missing rate.

Based on detection 1 and 4, we could have a reliable estimation of where the cell should be at time t+1 and retrieve it. The procedure is similar to our seed-based cell territory refinement. In practice, the seed region is set as the intersection between the parent detection and kid detection at the frame that missing happens.

It is worthy to note that the two modules in our framework focus on different aspects to refine the cell detection and segmentation results. To avoid making the system cumbersome, we did not allow jump when dealing with under-segmentation and over-segmentation problems in module one. Similarly, we did not allow detections to split or merge in module two when dealing with cell missing.

Step 5: Post-processing

Tracklets association

In the iterative tracking and error correction stages, we control a reasonable FDR when designing the costs in Equation 20, while untracked outliers always exist and can lead to broken tracklets. To completely reconstruct the lineage, we execute the track stage one more time but consider the head and tail of a track only. Because most of the reliable motions have been successfully associated, the new distance distributions considering heads and tails only can have a larger variance, and hence the outliers with longer motions can be associated under the control of the same FDR. This step usually eliminates false negative associations without bringing more false positives since extra information from tracking results is utilized.

bioRxiv preprint doi: https://doi.org/10.64898/2026.03.12.711203; this version posted March 16, 2026. The copyright holder for this preprint (which was not certified by peer review) is the author/funder, who has granted bioRxiv a license to display the preprint in perpetuity. It is made available under aCC-BY 4.0 International license.

If a user would rather further complete the cell lineage than improve the tracking performance, another option is to associate cells compulsorily. In this way, tracklets are associated as far as possible under some loose conditions, such as the maximal motion distance. It can help some high-level biological researches that may not be so sensitive to tracking accuracy, such as the embryonic development at the tissue and organ level.

Division detection

Division detection is a even more tough task than cell tracking because of several reasons. First of all, it refers to one-to-multiple matchings, which is more difficult and cannot be included in most tracking methods. Secondly, the cell morphology has a great transition after division, which leads to a lower affinity score or matching probability. And the most important reason is that division detection has a extremely high accuracy requirement. For example, cells in one embryonic data has a 1% probability to divide and the accuracy of a division detection algorithm is 99%. In this case, the final reconstruction result cannot benefit from introducing the algorithm at all since it will bring more errors (1%) than corrections (0.99%).

Therefore, in our pipeline, divisions are not processed together with normal cell tracking but in the post-processing module (Fig. 1F). The philosophy behind is to focus on the key problem at once, where the association of the majority cell transitions can be processed first and filtered, and then a much smaller number of detection candidates can tolerate more errors but maintain the same performance. Besides, another advantage of this arrangement is that division detection can learn more information from the tracking results as well. For example, if the tail of one track is adjacent to two heads of other tracks in the next time point, it is likely to be a division. However, if all the three tracks are long and stable, the probability of being a division is larger than three short and unstable tracks, because the reason causing broken can be multifarious for the latter, such as bad data quality. Benefiting from the robust tracking algorithm and high-accurate results, it is possible to detect most divisions only using some simple rule-based decision tree.

There are three major rules to distinguish division and normal cells.

Rule 1 Every cell nucleus is a dense ball, but the nuclei group is sparse. We assume the fluorescent imaging of a cell nucleus is dense without too much texture or gaps inside, and is not touched with its neighbors since nuclei are usually at the center of a cell. Hence, if two nuclei are touched, manifested as one segmented instance with two isolated and similar components, they are very likely to be cells in division. However, since the correctness of the assumption varies between data, where some imaging data has stripe artifacts and cause gaps in nuclei, the rule is optional and may not work on all data.

Rule 2 If a stable track starts with a small-volume cell as the head, it is very likely to be a cell in division. A stable track means the track length is long enough, which implies the guarantee of the data quality and small probability to meet a broken. If the size of the head cell of the track is half of that of its candidate parent, the probability of division is also very high. Since a division always brings a new track, this rule works for most divisions if segmentation and tracking are good enough (Fig. S9B).

Rule 3 The migration of cells in the field of view follows Brownian motion with drift (Fig. S9C). During cell division, two new cells usually carry momentum of the same size but opposite direction, manifested as rapid movement of the two cells in opposite directions. If candidate cells move in the same direction, it is almost impossible for true cell division to occur. We use this information and assume that the

bioRxiv preprint doi: https://doi.org/10.64898/2026.03.12.711203; this version posted March 16, 2026. The copyright holder for this preprint (which was not certified by peer review) is the author/funder, who has granted bioRxiv a license to display the preprint in perpetuity. It is made available under aCC-BY 4.0 International license.

coordinates of the dividing cells in a certain frame are B_0, and the coordinates of the two candidate offspring cells are B_{11}, B_{12}, respectively, then we have:

$$B_{11} - B_0 \sim N\left(\mu_{11} + d, \frac{1}{n-1}\sum_{i=1}^{n}(B_i - \mu_i)^2\right) \tag{25}$$

$$B_{12} - B_0 \sim N\left(\mu_{12} - d, \frac{1}{n-1}\sum_{i=1}^{n}(B_i - \mu_i)^2\right) \tag{26}$$

Among them, d is an additional drift caused by cell division. Due to the uncertainty of cell center markers and the influence of cell size during cell segmentation, the center position of cells follows the following distribution:

$$C \sim N(0, (\frac{1}{3}R)^2) \tag{27}$$

Taking into account the impact of the above two factors, there are

$$x_{11} + x_{12} \sim N(\mu_{11} + \mu_{12}, \frac{2}{n-1}\sum_{i=1}^{n}(B_i - \mu_i)^2 + (\frac{1}{3}R_1)^2 + \left(\frac{1}{3}R_2)^2\right) \tag{28}$$

Note that $x \in R^3$, assuming that each dimension follows the above distribution, then:

$$\sum_{k=1}^{3}\left(\frac{(x_{11}^k + x_{12}^k) - (\mu_{11}^k + \mu_{12}^k)}{\sqrt{\frac{2}{n-1}\sum_{i=1}^{n}(B_i^k - \mu_i^k)^2 + (\frac{1}{3}R_1)^2 + (\frac{1}{3}R_2)^2}}\right)^2 \sim \chi_3^2 \tag{29}$$

Based on the three rules, we design a decision tree (Fig. S9A). For every cell, first of all, we will check it has two isolated components or not, and then check if it is the head or second head of a track and satisfy several requirements simultaneously, such as track length and size ratio. Then we will try the best to assign the most matched parent or children if it passes all examinations.

Multi-angle stitching

To capture the whole embryo activity, some types of microscopes, such as light-sheet microscopes, records the specimen from different angles simultaneously, where cells may move out of the field of one view. There are two techniques to fully reconstruct the lineage: fusing the images or stitching the tracks. We adopted the latter for several reasons. Firstly, the image resolution is anisotropic, where the axial resolution is usually 3 to 10 times worse than the lateral resolution. Fusing images from different angles requires upsampling, means a huge increase of data size, and brings more pressure on memory and storage, but there is no information gain. Secondly, if one cell is recorded in multiple images, the data qualities are usually different, where the reason of multi-angle imaging is to guarantee good data quality in at least one image. Image fusion will average the data qualities, which is always worse than tracking the best one.

Oppositely, stitching tracks can not only consume nearly negligible computation and storage resources compare to image fusion but also make the best of all angles. We can get the approximate

bioRxiv preprint doi: https://doi.org/10.64898/2026.03.12.711203; this version posted March 16, 2026. The copyright holder for this preprint (which was not certified by peer review) is the author/funder, who has granted bioRxiv a license to display the preprint in perpetuity. It is made available under aCC-BY 4.0 International license.

location correspondence after the spatial registration between all angles utilizing the approach discussed before. If a cell is highly-overlapped with another cell in a different angle, they are regarded as the same one. Then if two tracks have consecutively highly-overlapped cells, they are regarded as the same tracked and merged. Non-overlapped cells are preserved as much as possible, while long enough but not overlapped tails are also considered as divisions.

bioRxiv preprint doi: https://doi.org/10.64898/2026.03.12.711203; this version posted March 16, 2026. The copyright holder for this preprint (which was not certified by peer review) is the author/funder, who has granted bioRxiv a license to display the preprint in perpetuity. It is made available under aCC-BY 4.0 International license.

Figures S1 to S15

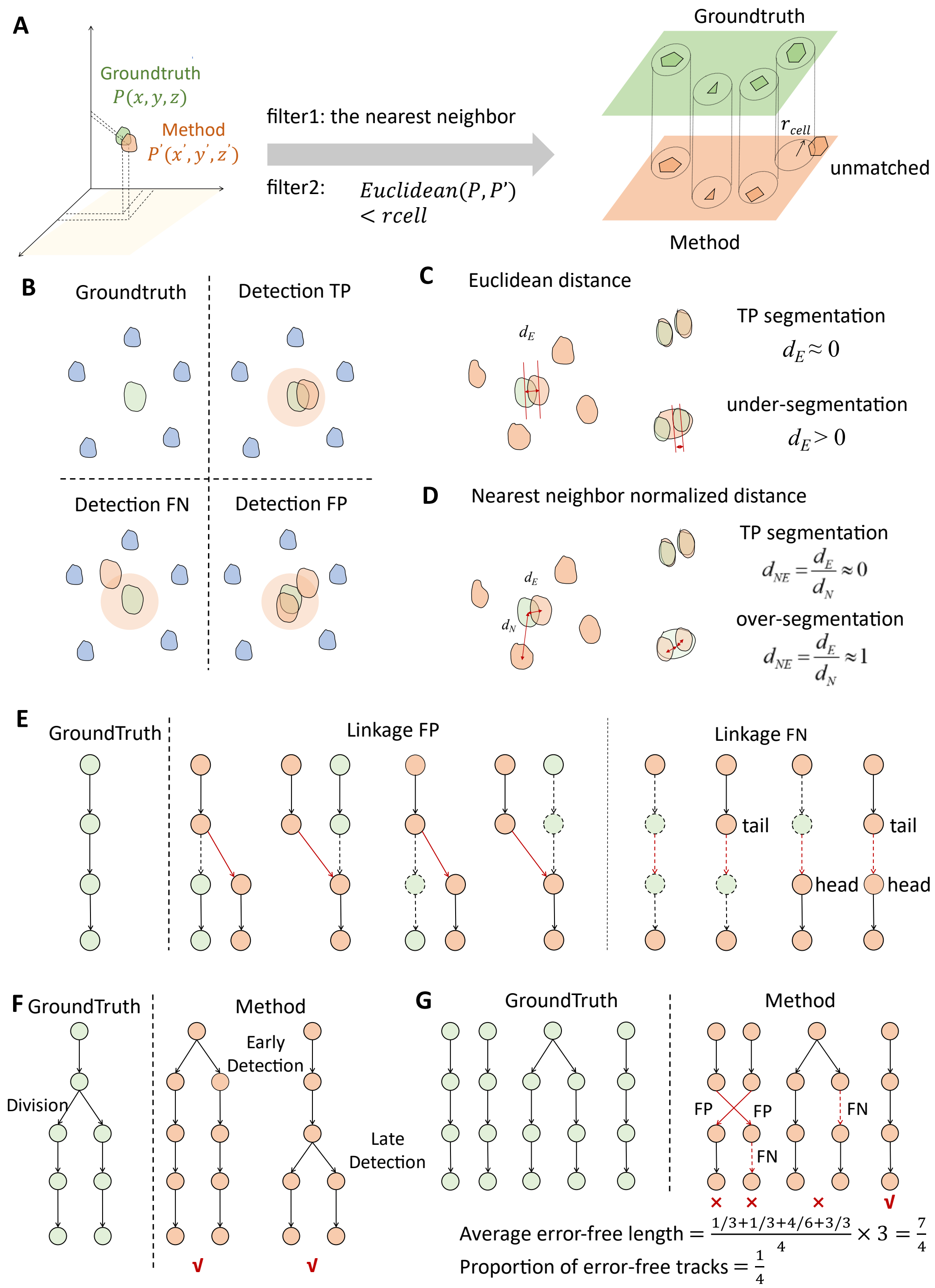

Fig. S1 Evaluation metrics of cell tracking.

(A) Cell matching between ground truth and method results. A cell detection is considered correct when a cell in the ground truth and a cell in the method results have an Euclidean distance that is less than the cell radius and are the nearest neighbors of each other.

(B) Illustration of TP, FP, and FN cases in cell detection. A detection is considered TP when there is exactly one detected cell within a certain distance range of a ground truth cell. If there are more cells in this range, the

bioRxiv preprint doi: https://doi.org/10.64898/2026.03.12.711203; this version posted March 16, 2026. The copyright holder for this preprint (which was not certified by peer review) is the author/funder, who has granted bioRxiv a license to display the preprint in perpetuity. It is made available under aCC-BY 4.0 International license.

excess cells are classified as FP errors. If there is less than one detected cell (i.e., none), it is classified as a FN error.

(C) Euclidean distance metric for cell detection. This metric refers to the average of the nearest distances from each cell in the ground truth to any cell in the method results. If cell detection is completely correct, this metric is close to 0; if significant under-segmentation occurs, this metric will be large.

(D) Normalized Nearest Neighbor Distance metric for cell detection. This metric is the Euclidean distance metric normalized by the average distance to adjacent cells in the method results. If cell detection is completely correct, this metric is close to 0; if significant over-segmentation occurs, this metric will approach 1.

(E) Illustration of FP and FN in cell tracking. For a link between two consecutive frames in the ground truth, if the cell at at least one end of the link is incorrectly connected to a different cell, it is classified as a FP error. If this link is missing, whether due to a missing cell detection or a track break, it is classified as a FN error.

(F) Evaluation correction for division events. Since cell division is a long-duration process and annotation errors may occur, we adopt a lenient evaluation strategy for divisions that occur earlier or later than in the ground truth, meaning they are not counted as FP or FN errors.

(G) Illustration of the calculation for average error-free length and proportion of error-free tracks. We define these two metrics to characterize the continuity of the tracks.

bioRxiv preprint doi: https://doi.org/10.64898/2026.03.12.711203; this version posted March 16, 2026. The copyright holder for this preprint (which was not certified by peer review) is the author/funder, who has granted bioRxiv a license to display the preprint in perpetuity. It is made available under aCC-BY 4.0 International license.

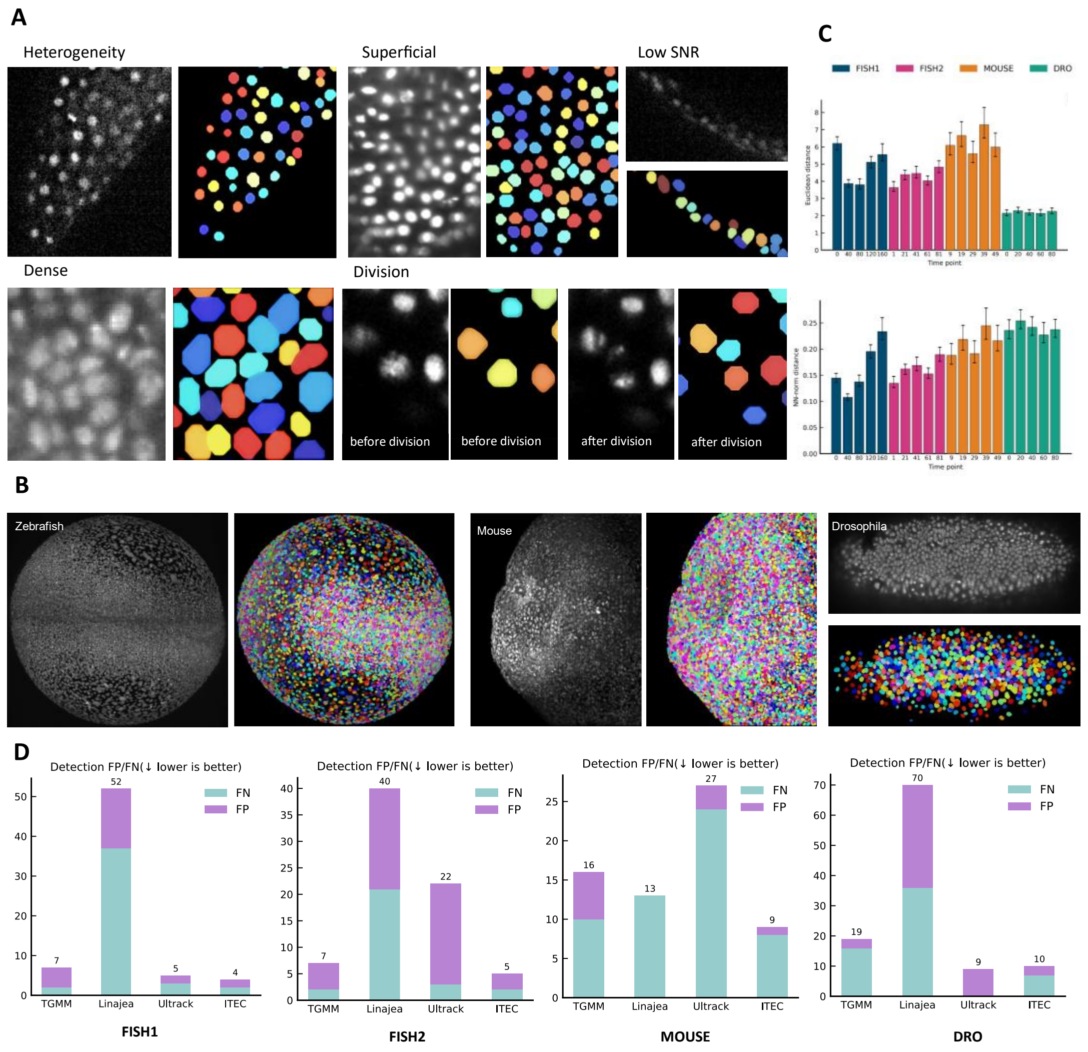

Fig. S2 Robust cell detection in complex scenarios.

(A) Local view of cell segmentation in different scenarios based on ITEC. ITEC achieves high-level segmentation under conditions of heterogeneity, superficial, low SNR, dense, and division.

(B) Projection of cell segmentation for different species based on ITEC. The raw image uses the maximum z-axis projection, while the segmented image uses the nearest z-axis projection.

(C) Average Euclidean distance (pixels) and normalized nearest-neighbor distance (error bars, 10th and 90th percentile confidence intervals). Both the Euclidean distance and the normalized nearest-neighbor distance are very low.

(D) Detection FP/FN of ITEC and peer methods based on 500, 500, 250, and 500 annotated cells, respectively on the four datasets.

bioRxiv preprint doi: https://doi.org/10.64898/2026.03.12.711203; this version posted March 16, 2026. The copyright holder for this preprint (which was not certified by peer review) is the author/funder, who has granted bioRxiv a license to display the preprint in perpetuity. It is made available under aCC-BY 4.0 International license.

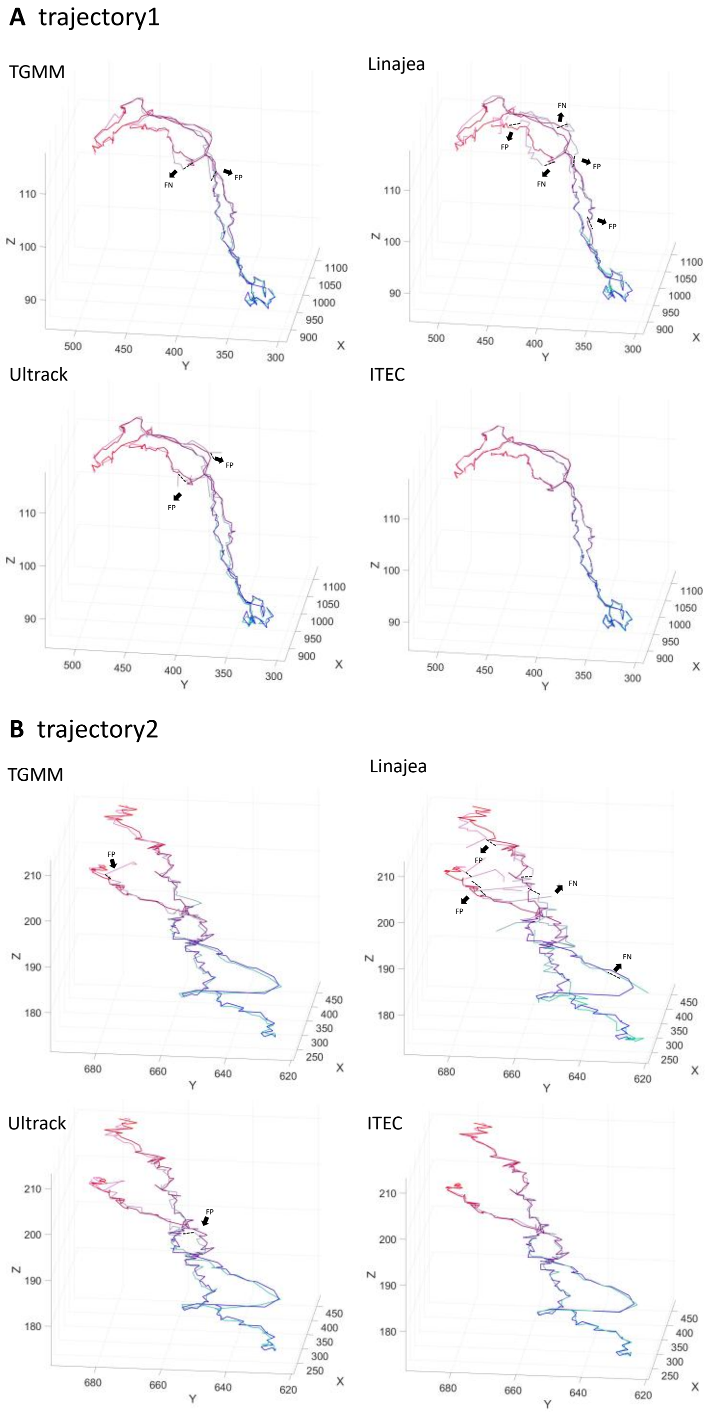

Fig. S3 Comparision of tracking errors between ITEC and three peer methods. Track 1 and Track 2 are derived from the FISH1 and FISH2 datasets, respectively. Lines ranging from blue to red represent the ground truth, while lines ranging from cyan to magenta represent the algorithm results. Erroneous tracks are marked with dashed lines. Only the closest points of the algorithm's coordinate points that match the ground truth are displayed.

bioRxiv preprint doi: https://doi.org/10.64898/2026.03.12.711203; this version posted March 16, 2026. The copyright holder for this preprint (which was not certified by peer review) is the author/funder, who has granted bioRxiv a license to display the preprint in perpetuity. It is made available under aCC-BY 4.0 International license.

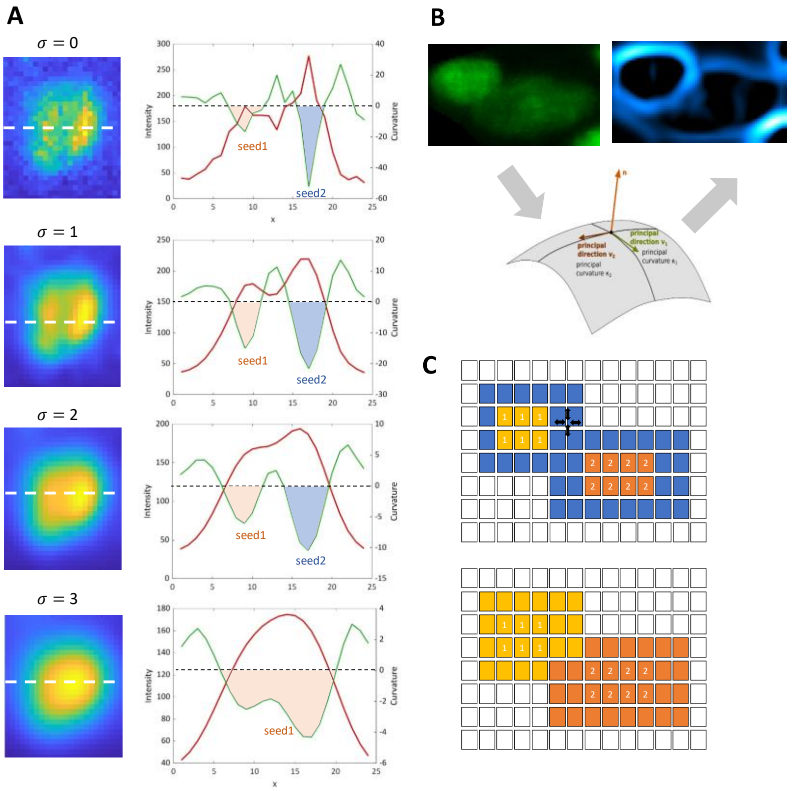

Fig. S4 Some principles of ITEC cell segmentation

(A) The high-order information of a typical cell, showing changes in cell brightness and curvature at different filtering scales. When the filtering scale is small, the cell is over-segmented into two cells. When the filtering scale is large, the over-segmentation is resolved, and the intensity of cell has only one peak.

(B) Raw data and its curvature map. On the left is the original data, where two cells are squeezed together and it is difficult to determine their boundaries through simple grayscale information; On the middle is the geometric representation of principal curvature. On the right is the curvature map, and the boundary is clearly visible.

(C) Seed regions. The yellow and orange colors on the left represent the seeds of two cells in (B), the blue color represents the area to be grown, and the right figure represents the well grown boundary.

bioRxiv preprint doi: https://doi.org/10.64898/2026.03.12.711203; this version posted March 16, 2026. The copyright holder for this preprint (which was not certified by peer review) is the author/funder, who has granted bioRxiv a license to display the preprint in perpetuity. It is made available under aCC-BY 4.0 International license.

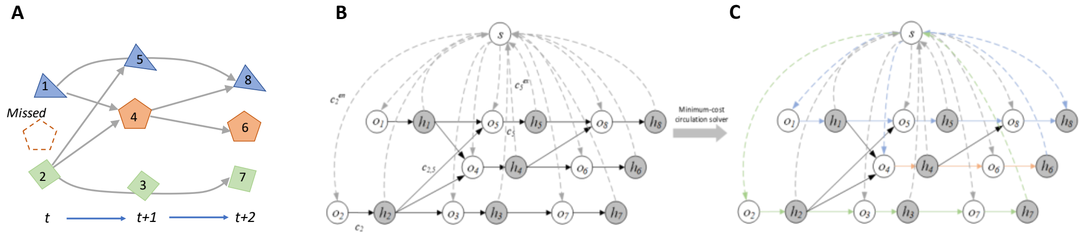

Fig. S5 Minimum-cost circulation framework

(A) Objects detected across three consecutive frames. The first frame includes two detections, with one missed detection indicated by orange dashed lines. The lines connecting detections represent possible associations, each assigned a certain cost. In total, three distinct tracks span these frames. For instance, detections 2, 3, and 7 belong to the same track and should be linked together.

(B) The minimum-cost circulation formulation for MOT problem. Each detection x_i is represented by two nodes: a pre-node o_i and a post-node h_i. A dummy node s is connected to all pre-nodes, and all post-nodes are linked back to s; these edges are shown as dashed lines. In this circulation network, flow conservation is maintained at every node, ensuring the dummy node's excess flow remains zero.

(C) The results from the proposed minimum-cost circulation framework. Three tracks are formed and they are shown with the same color as in (A).

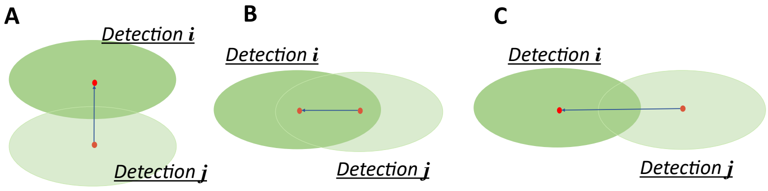

Fig. S6 Illustration of the drawbacks of overlapping ratio and center distance. Here detection i and j are adjacent cell regions at different time points. If we want to measure their similarity, the desired score should be able to discriminate these three conditions as similarity (B) > (A) > (C). If we measure them using overlapping ratio, (A) and (C) become equal, while (A) and (B) will be equal if we use center distance.

bioRxiv preprint doi: https://doi.org/10.64898/2026.03.12.711203; this version posted March 16, 2026. The copyright holder for this preprint (which was not certified by peer review) is the author/funder, who has granted bioRxiv a license to display the preprint in perpetuity. It is made available under aCC-BY 4.0 International license.

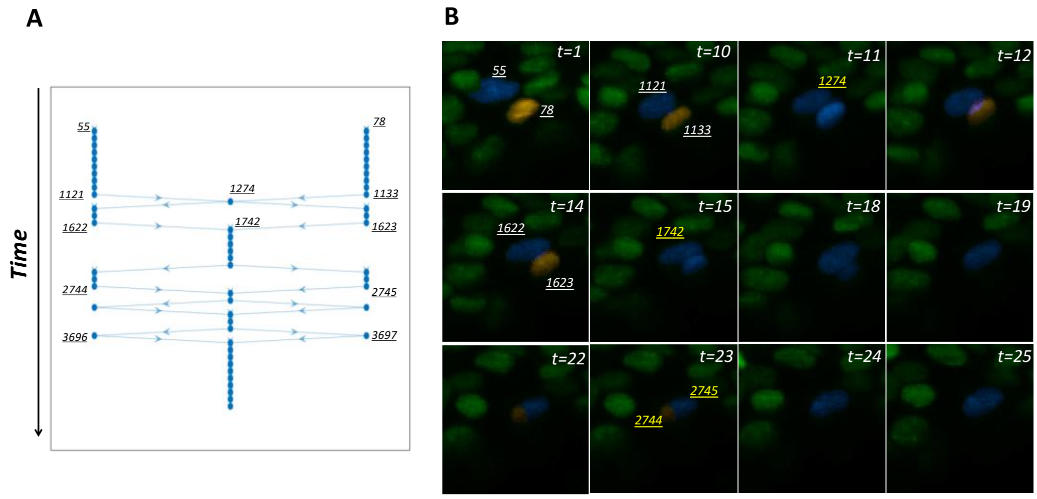

Fig. S7 Example of over segmentation/under segmentation correction module

(A) A typical error track. Each node represents a detection, and there have been several instances of over segmentation and under segmentation errors in the track. Our method will add one to two or two to one connections before and after nodes 1274, 1742, etc., and make subsequent judgments.

(B) The original image corresponding to this example. The typical segmentation errors (yellow ids) and the corresponding segmentation results in adjacent frames. Totally two cells are involved in this track. One of them fades away at time point 18-19.

bioRxiv preprint doi: https://doi.org/10.64898/2026.03.12.711203; this version posted March 16, 2026. The copyright holder for this preprint (which was not certified by peer review) is the author/funder, who has granted bioRxiv a license to display the preprint in perpetuity. It is made available under aCC-BY 4.0 International license.

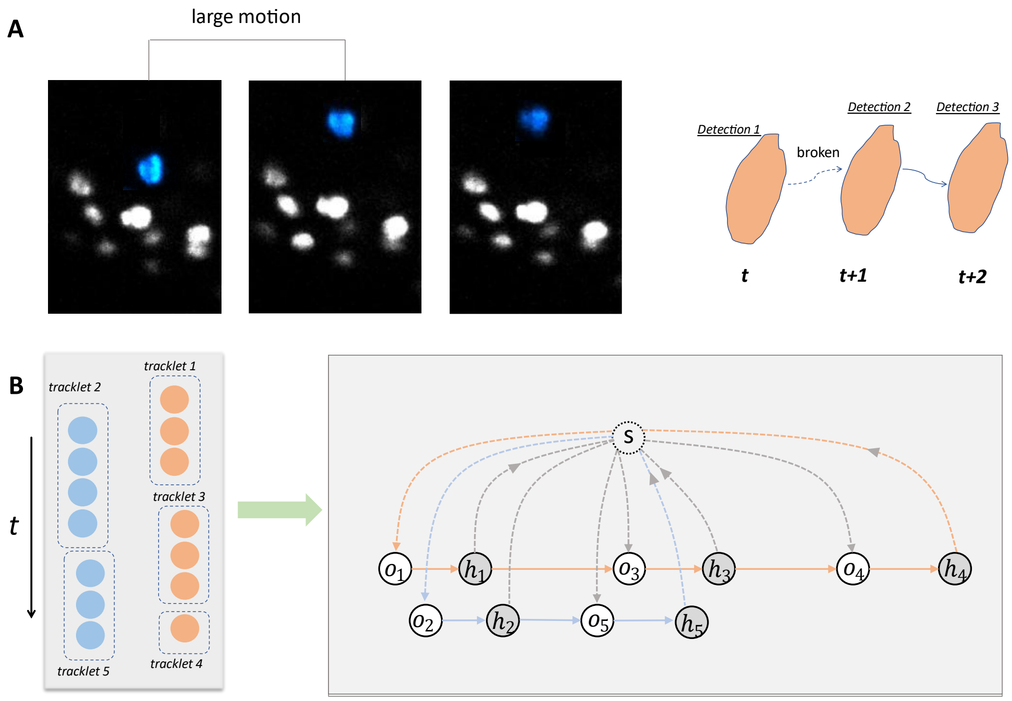

Fig. S8 Tracklets association.

(A) Typical track fracture. Due to the possibility of large-scale movement of cells, some associations may be disconnected during initial tracking.

(B) Tracklets association network. We update the association cost based on the tracklets and use the minimum-cost circulation framework for new association.

bioRxiv preprint doi: https://doi.org/10.64898/2026.03.12.711203; this version posted March 16, 2026. The copyright holder for this preprint (which was not certified by peer review) is the author/funder, who has granted bioRxiv a license to display the preprint in perpetuity. It is made available under aCC-BY 4.0 International license.

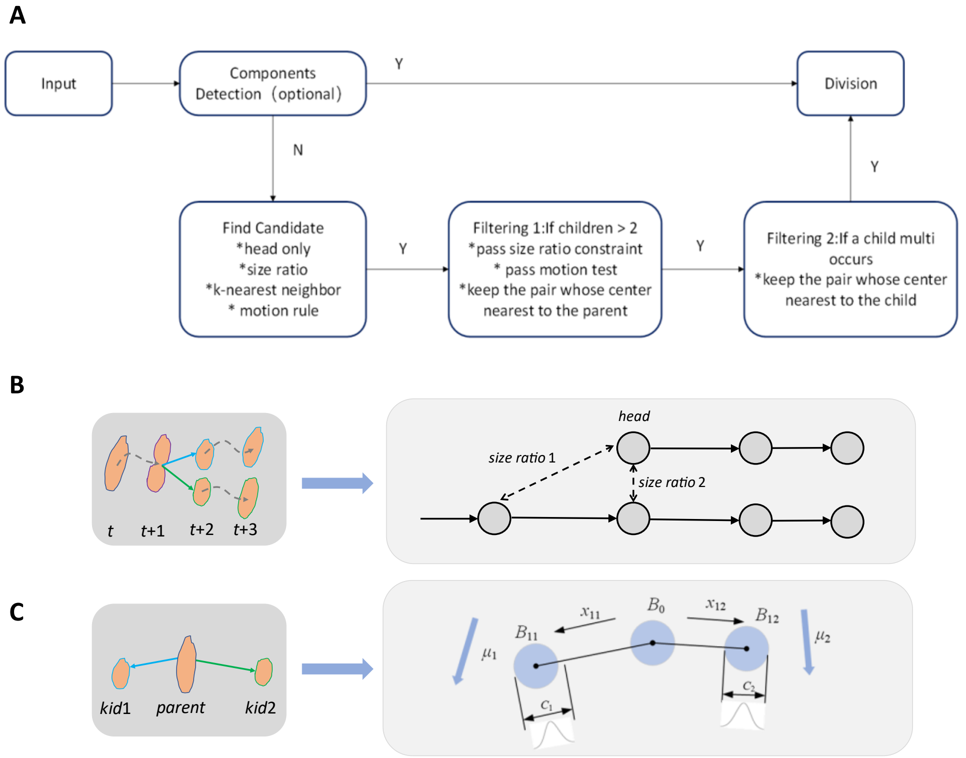

Fig. S9 Principle of cell division module.

(A) The pipeline of division detection. Firstly, for every cell segmentation result, it is reasonable but optional to treat a segmentation with two isolated components as divisions, under the assumption that cells are dense and integral. Then, for every candidate children, we will test it is a possible division or not. A candidate children must be the head of a track and satisfy the size ratio and motion rule requirements. It also need to be one of the nearest neighbor of its parents. For one parent, it is possible to have more than two candidate children. If so, we will check if there is a gap between any pair of two children, and only keep the pair whose center is nearest to the parent. Certainly, one child may also have multiple candidates as well. If so, we will follow the same strategy and only keep the nearest parent. The two steps can find most divisions.

(B) Division candidate filtering. The child candidate needs to be the head of a fracture track and meet certain size constraints.

(C) An example of cell division. B_0 represents the parent cell, B_{11} and B_{12} represent the daughter cells. The two daughter cells have both the motion component caused by overall drift and the momentum generated by division.

bioRxiv preprint doi: https://doi.org/10.64898/2026.03.12.711203; this version posted March 16, 2026. The copyright holder for this preprint (which was not certified by peer review) is the author/funder, who has granted bioRxiv a license to display the preprint in perpetuity. It is made available under aCC-BY 4.0 International license.

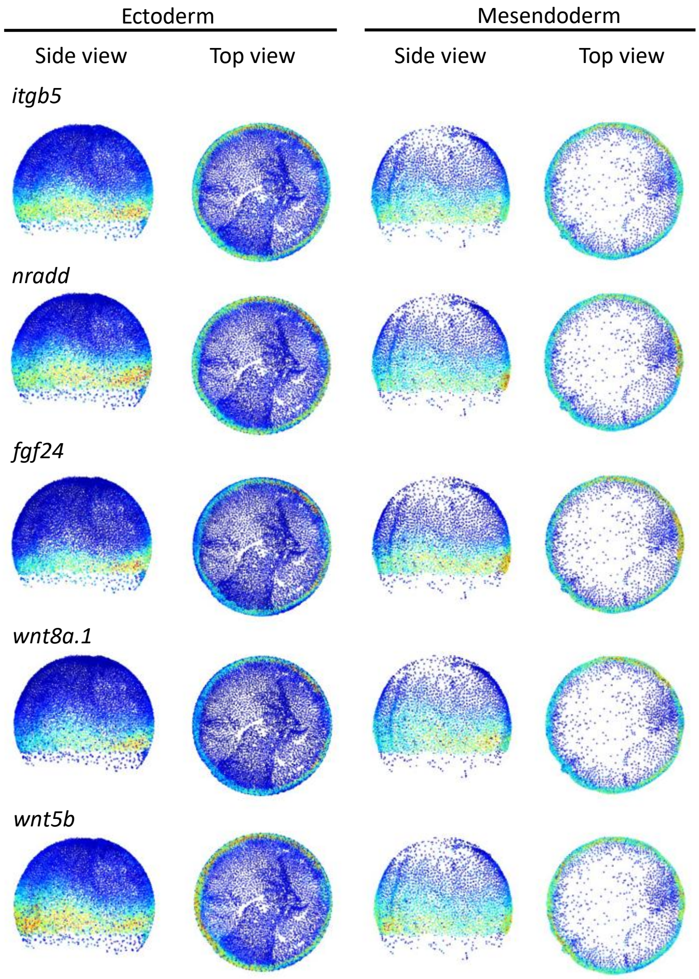

Fig. S10 The expression of the 5 genes most related to cell migration velocity.

bioRxiv preprint doi: https://doi.org/10.64898/2026.03.12.711203; this version posted March 16, 2026. The copyright holder for this preprint (which was not certified by peer review) is the author/funder, who has granted bioRxiv a license to display the preprint in perpetuity. It is made available under aCC-BY 4.0 International license.

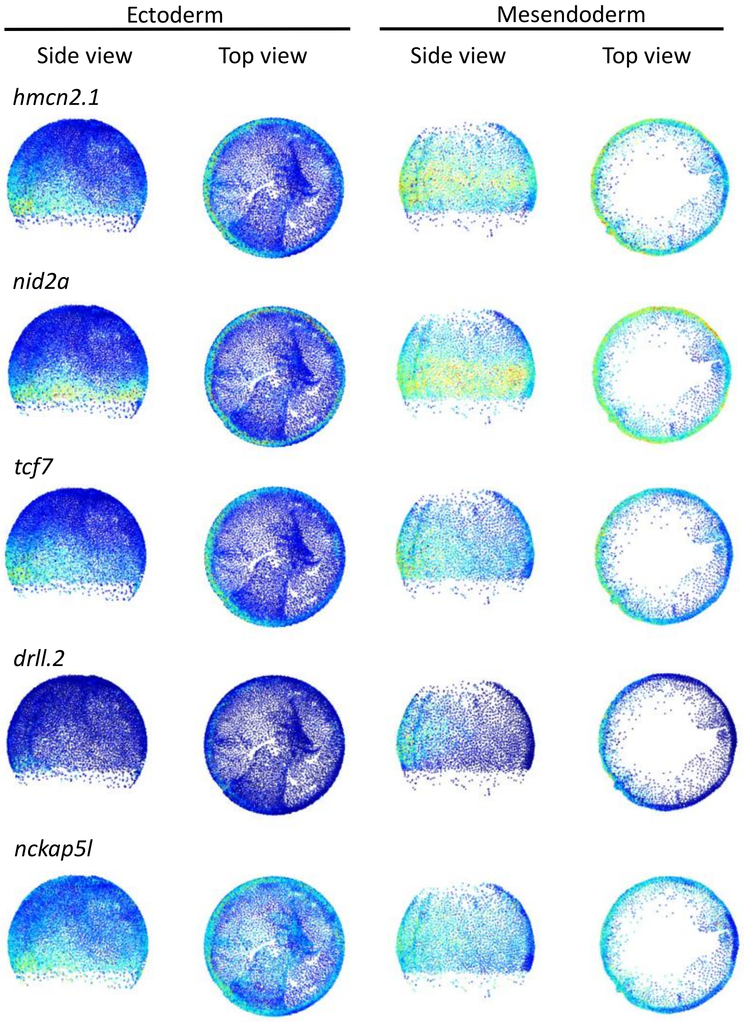

Fig. S11 The expression of the 5 genes most related to cell motion variance.

bioRxiv preprint doi: https://doi.org/10.64898/2026.03.12.711203; this version posted March 16, 2026. The copyright holder for this preprint (which was not certified by peer review) is the author/funder, who has granted bioRxiv a license to display the preprint in perpetuity. It is made available under aCC-BY 4.0 International license.

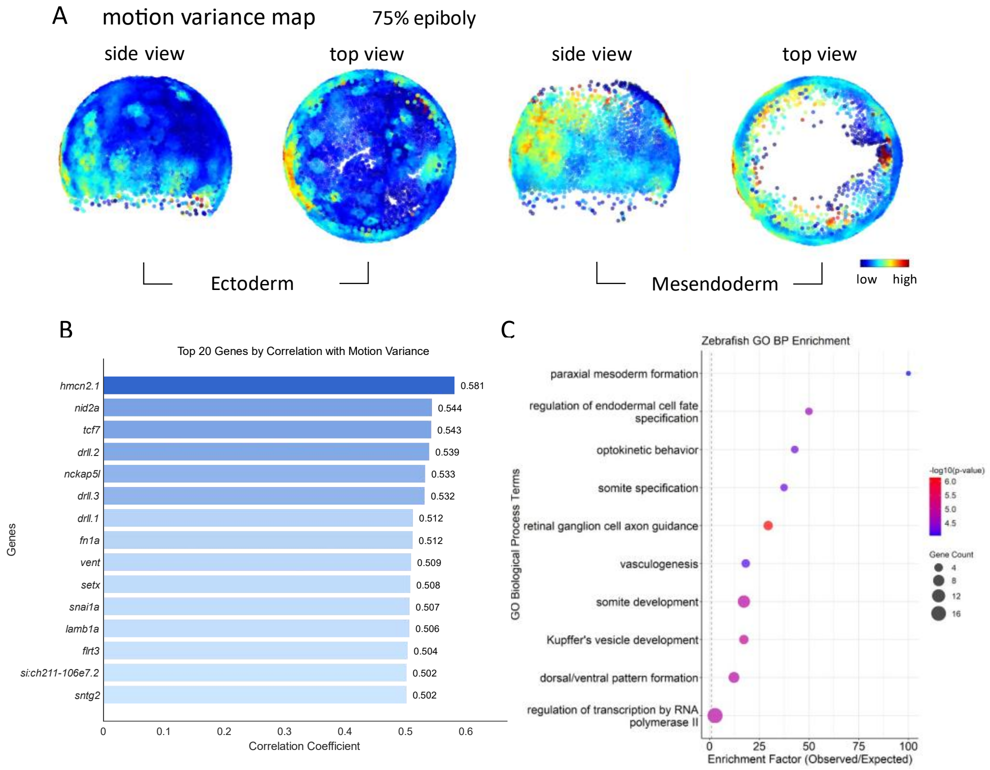

Fig. S12 Analyze of cell motion variance at 75% epiboly.

(A) Cell motion variance of ectoderm and mesoderm at 75% epiboly.

(B) GO term enrichment of top 90 genes positively correlated with motion variance.

(C) Top 20 genes most strongly positively correlated with motion variance and their correlation coefficients.

bioRxiv preprint doi: https://doi.org/10.64898/2026.03.12.711203; this version posted March 16, 2026. The copyright holder for this preprint (which was not certified by peer review) is the author/funder, who has granted bioRxiv a license to display the preprint in perpetuity. It is made available under aCC-BY 4.0 International license.

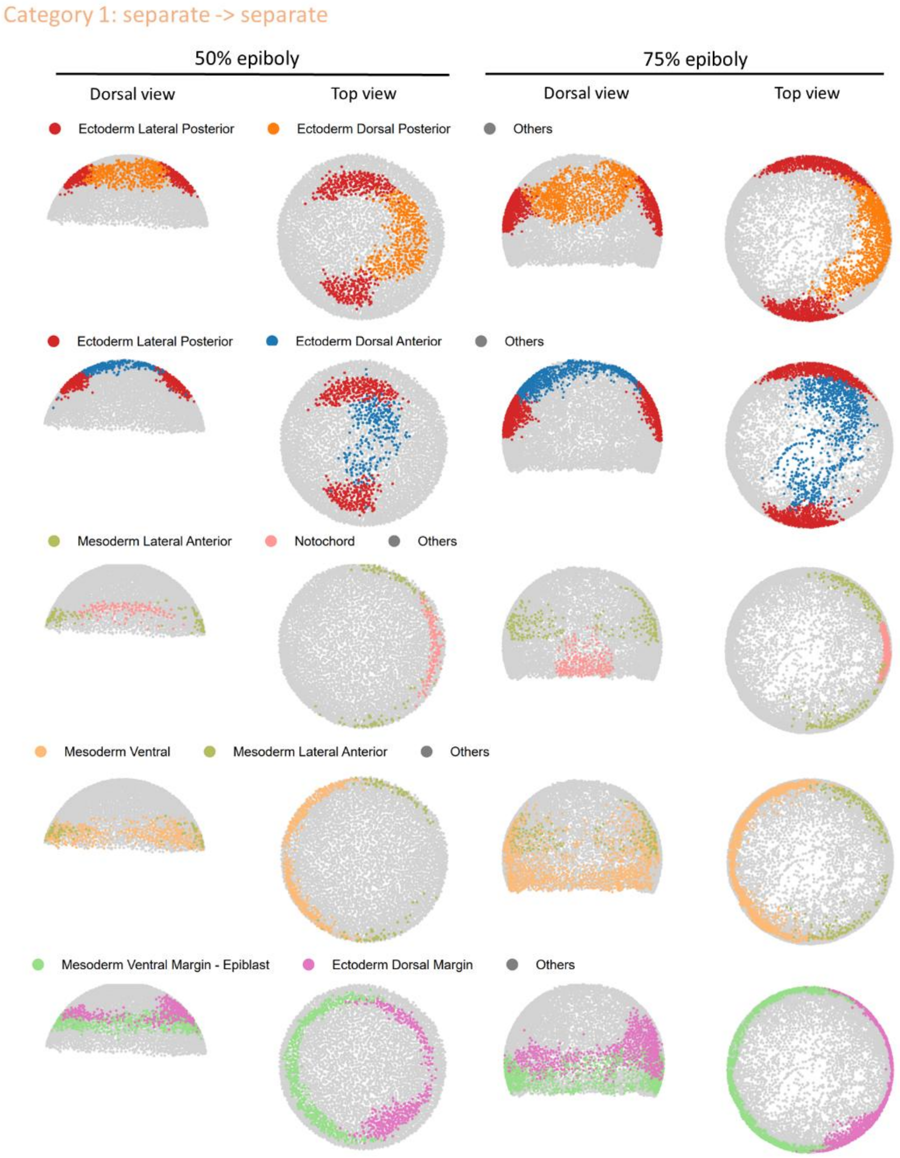

Fig. S13 Examples of separate category boundaries maintained from 50% to 75% epiboly.

bioRxiv preprint doi: https://doi.org/10.64898/2026.03.12.711203; this version posted March 16, 2026. The copyright holder for this preprint (which was not certified by peer review) is the author/funder, who has granted bioRxiv a license to display the preprint in perpetuity. It is made available under aCC-BY 4.0 International license.

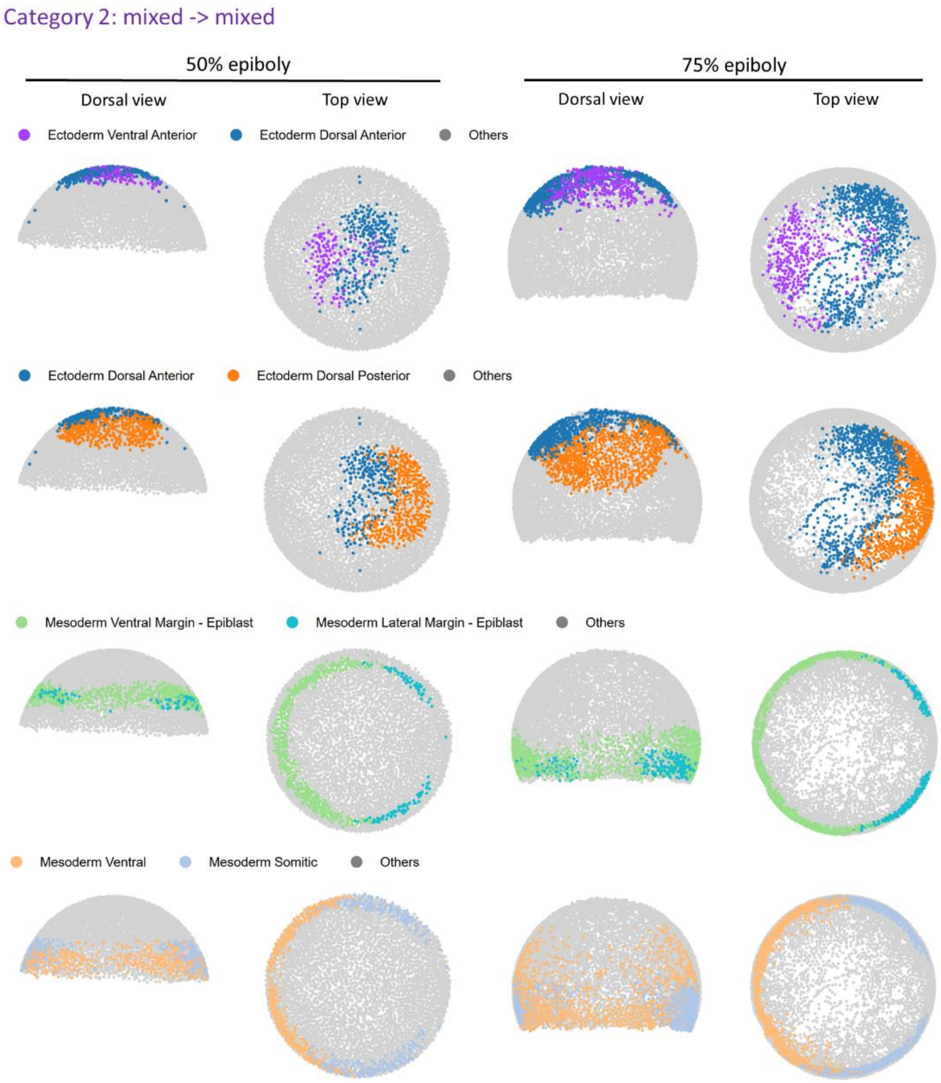

Fig. S14 Examples of persistently mixed category boundaries from 50% to 75% epiboly.

bioRxiv preprint doi: https://doi.org/10.64898/2026.03.12.711203; this version posted March 16, 2026. The copyright holder for this preprint (which was not certified by peer review) is the author/funder, who has granted bioRxiv a license to display the preprint in perpetuity. It is made available under aCC-BY 4.0 International license.

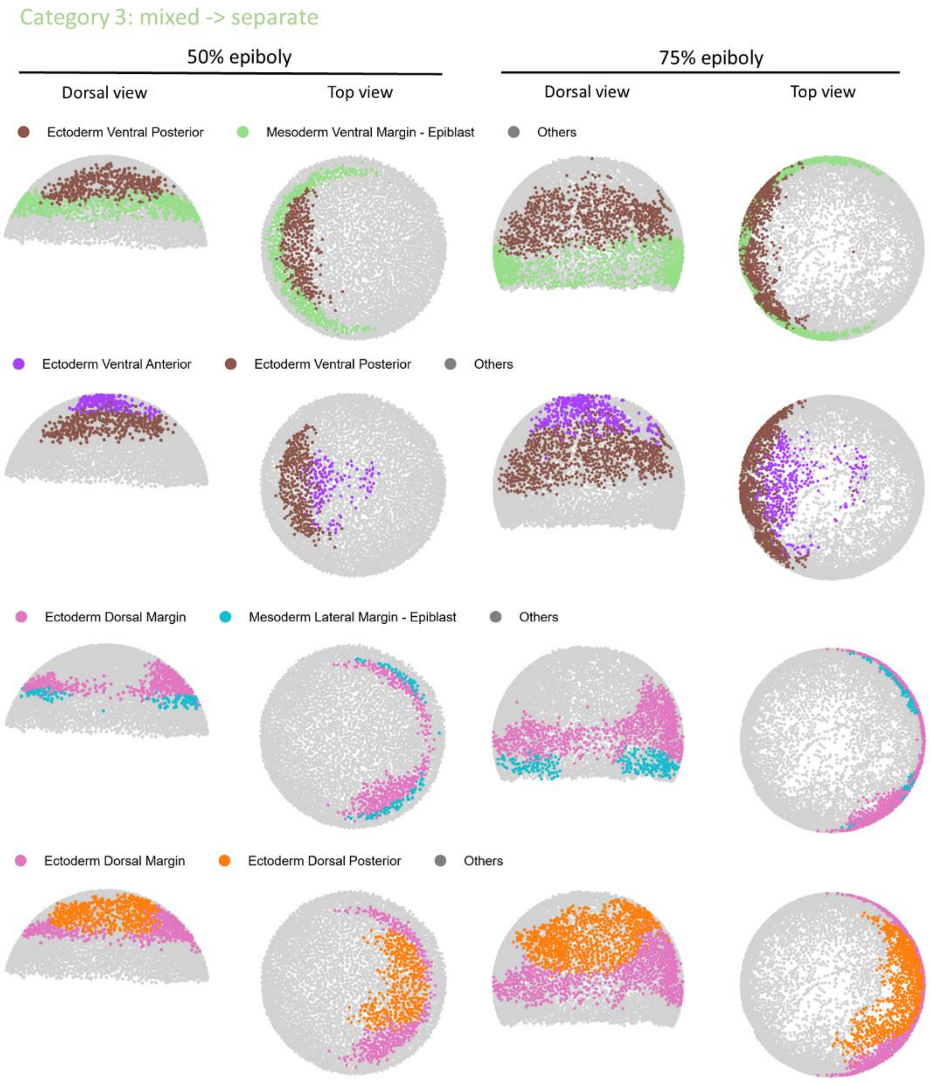

Fig. S15 Examples of category boundaries transitioning from mixed to separate between 50% to 75% epiboly.

bioRxiv preprint doi: https://doi.org/10.64898/2026.03.12.711203; this version posted March 16, 2026. The copyright holder for this preprint (which was not certified by peer review) is the author/funder, who has granted bioRxiv a license to display the preprint in perpetuity. It is made available under aCC-BY 4.0 International license.

Tables S1 to S3

Table S1 Peer method comparison

Methods	Training	Detection	Tracking
TGMM	Unsupervised	Watershed	Gaussian mixture model
Linajea	Supervised	U-Net	U-Net + ILP
Ultrack	Unsupervised	Multiple	UCM + ILP

Table S2 Embryonic development datasets and annotations

Name	Animal	Image size	Time point	Cell (roughly)	Annotation
FISH1	Zebrafish	1920 × 1920 × 180	192	1,590k	32, 946
FISH2	Zebrafish	1818 × 1792 × 253	100	1,575k	6, 841
MOUSE	Mouse	2169 × 2048 × 988	50	815k	2, 345
DRO	*Drosophila*	730 × 320 × 30	100	66k	2, 423

Table S3 Some parameters in ITEC pipeline

Parameters	Meanings
Min cell size	The volume lower bound of cells, unit in voxels
Max cell size	The volume upper bound of cells, unit in voxels
z-x/y ratio	The ratio of z-direction resolution to x/y direction resolution
Downsampling ratio	The downsampling ratio in the x/y direction (for acceleration)
Filter factor	The standard deviation of the Gaussian filter used for smoothing.
Background intensity	A general threshold of the background grayscale. Cells with intensity below the threshold won't be detected
Intensity upper bound	Pixels whose grayscale is above that bound will be set to that bound to ensure the contrast between pixels
Intensity lower bound	Minimum intensity of pure background
Intensity difference	Controls segmentation based on intensity difference between the cells and background
Curvature threshold	The threshold of detecting seeds for core regions
Foreground threshold	The threshold of detecting boundaries
Max Iteration	The max number of iteration steps of error correction
Division threshold	The confidence level for division detection
Max distance	a rough bound of the maximum displacement in pixels from frame t to t+1

Movies S1 to S5

bioRxiv preprint doi: https://doi.org/10.64898/2026.03.12.711203; this version posted March 16, 2026. The copyright holder for this preprint (which was not certified by peer review) is the author/funder, who has granted bioRxiv a license to display the preprint in perpetuity. It is made available under aCC-BY 4.0 International license.

Please see https://cloud.tsinghua.edu.cn/d/02fbca1ceceb490b814d/.

Movie S1 An example of cell tracking using ITEC

Movie S2 High-accuracy tracking using ITEC

Movie S3 Long-term lineage reconstruction of zebrafish embryo

Movie S4 Application of ITEC: Fate mapping

Movie S5 Application of ITEC: Revealing the zebrafish somitogenesis
